# Distinct Motifs in ATAD5 C-Terminal Domain Modulate PCNA Unloading Process

**DOI:** 10.3390/cells11111832

**Published:** 2022-06-03

**Authors:** Eunjin Ryu, Na Young Ha, Woojae Jung, Juyeong Yoo, Kyungjae Myung, Sukhyun Kang

**Affiliations:** 1 Center for Genomic Integrity, Institute for Basic Science, Ulsan 44919, Korea; rej911@unist.ac.kr (E.R.); hany8378@ibs.re.kr (N.Y.H.); dnwo0504@unist.ac.kr (W.J.); dwbndud1210@unist.ac.kr (J.Y.); kmyung@ibs.re.kr (K.M.); 2 Department of Biological Sciences, Ulsan National Institute of Science and Technology, Ulsan 44919, Korea; 3 Department of Biomedical Engineering, Ulsan National Institute of Science and Technology, Ulsan 44919, Korea

**Keywords:** ATAD5, PCNA, PCNA unloading, RFC-like complex, RLC, SUMOylation, RFC

## Abstract

Proliferating cell nuclear antigen (PCNA) is a DNA clamp that functions in key roles for DNA replication and repair. After the completion of DNA synthesis, PCNA should be unloaded from DNA in a timely way. The ATAD5-RFC-Like Complex (ATAD5-RLC) unloads PCNA from DNA. However, the mechanism of the PCNA-unloading process remains unclear. In this study, we determined the minimal PCNA-unloading domain (ULD) of ATAD5. We identified several motifs in the ATAD5 ULD that are essential in the PCNA-unloading process. The C-terminus of ULD is required for the stable association of RFC2-5 for active RLC formation. The N-terminus of ULD participates in the opening of the PCNA ring. ATAD5-RLC was more robustly bound to open-liable PCNA compared to the wild type. These results suggest that distinct motifs of the ATAD5 ULD participate in each step of the PCNA-unloading process.

## 1. Introduction

Proliferating cell nuclear antigen (PCNA) is a DNA clamp [1]. PCNA is loaded onto the primer-template junction and recruits DNA polymerase δ to initiate DNA synthesis, especially for lagging strands [2,3]. PCNA encircles DNA and tethers DNA polymerases to enhance processivity. PCNA forms a homotrimeric, closed-ring structure. Therefore, opening and closing the PCNA ring is required for the DNA loading of PCNA [4]. The loading process is mediated by clamp-loader complexes [5,6,7]. Replication Factor C (RFC) is a major PCNA-loading clamp-loader-complex. RFC is a pentameric complex, which is composed of RFC1-5. RFC opens an interface between PCNA subunits and loads PCNA onto primer-template DNA or nicked DNA. Eukaryotic cells have other clamp-loader complexes called RFC-Like Complexes (RLCs) [8,9]. RFC and RLCs share four small subunits, RFC2-5, and the largest subunit of each complex determines its function. CTF18-RLC contains a CTF18-DCC1-CTF8 trimer instead of RFC1, and functions as a PCNA-loading complex for the leading strand synthesis [10]. 

After the completion of DNA synthesis, PCNA is released from the DNA. Because PCNA forms a closed-ring structure that encircles DNA, the spontaneous release of PCNA from DNA is slow, unless it is actively removed from DNA [11,12]. The prolonged accumulation of PCNA on the chromatin causes various problems. PCNA interacts with DNA replication and repair factors and functions as a molecular hub for chromosome duplication and DNA repair [3]. Inappropriately retained PCNA on chromatin recruits unnecessary proteins and causes damage to the genome [13,14,15,16,17,18]. In addition, PCNA should be rapidly recycled from the replicated DNA to the next primer-templated junctions, considering the large amount of Okazaki fragments synthesized during the S phase. If not, DNA replication is delayed, and under-replicated regions can be formed [11]. Therefore, an active PCNA-unloading machinery is required for efficient DNA replication. In eukaryotic cells, ATAD5-RLC functions as a PCNA-unloading complex [11]. ATAD5-RLC consists of ATAD5 and RFC2-5. Previously, we demonstrated that ATAD5-RLC possesses unique PCNA-unloading activity [11,19]. ATAD5 is primarily composed of two domains. The N-terminal domain, ATAD5 (1–600), contains binding motifs for Ubiquitin Specific Proteases (USPs) and facilitates the de-ubiquitination of Ub-PCNA [15]. The C-terminal domain, ATAD5 (601–1844), has an AAA+ ATPase domain and a binding site for RFC2-5. Two interfaces between ATAD5 and RFC2-5, the ATPase motif and collar domain, are crucial for PCNA-unloading activity [11]. Single-molecule experiments have demonstrated that ATAD5-RLC binds to DNA-loaded PCNA and opens the PCNA ring. Then, the PCNA-ATAD5-RLC intermediate is released from the DNA. However, the way PCNA unloading is driven by ATAD5-RLC remains to be elucidated [11].

In this study, we attempted to understand the PCNA-unloading process by examining the functions of several conserved motifs in the ATAD5 C-terminal domain. We determined the minimal PCNA-unloading domain (ULD) of ATAD5. The upper region of the ULD is required for an efficient PCNA ring opening. ATAD5-RLC bound more stably to the open-liable PCNA mutant than to wild-type PCNA. The C-terminus of the ULD is important for stable RFC2-5 association with ATAD5. Our results showed that specific motifs in the ATAD5 ULD cooperatively open the PCNA ring for DNA release.

## 2. Materials and Methods

### 2.1. Protein Purification

ATAD5-RLCs were purified using the Bac-to-Bac Baculovirus expression system (Thermo Fisher Scientific, Waltham, MA, USA). Viruses were prepared using Sf9 cells, and proteins were expressed in Hi-5 cells. N-terminal 6× His-tagged and C-terminal 2× StrepII-3× FLAG-tagged ATAD5 (812–1844) were cloned into a pFastBac vector. N-terminal GST-3xHA-tagged PCNAs was expressed in *E. coli* BL21 (DE3) cells (Enzynomics, Daejeon, Korea).

The proteins were prepared as described previously [11]. PCNA expressing *E. coli* cells were lyzed in 0.5 M KCl Buffer H (25 mM HEPES [pH 7.5], 1 mM EDTA, 1 mM EGTA, 2.5 mM magnesium acetate, 10% glycerol, 1 mM ATP, and 0.02% NP40) using lysozyme and sonication. After clarification of lysate using centrifugation, PCNA was captured via GST Bind Agarose Resin (ELPISBIO, Daejeon, Korea) and eluted using precision protease digestion. PCNA was further purified using Q Sepharose. In the case of ATAD5, cells were resuspended in 300 mM potassium acetate [KoAc] Buffer H (25 mM HEPES [pH 7.5], 1 mM EDTA, 1mM EGTA, 2.5 mM magnesium acetate, 10% glycerol, 1 mM ATP, and 0.02% NP40, with 1× complete protease inhibitor cocktail (Merck, Darmstadt, Germany) and lyzed by sonication. The ATAD5-RLC complex was purified with the sequential application of cOmplete His-Tag Purification Resin (Merck, Darmstadt, Germany) and ANTI-FLAG^®^ M2 Affinity Gel (Merck, Darmstadt, Germany). Fractions containing ATAD5-RLC was pooled and applied to SP Sepharose. Purified proteins were analyzed using SDS-PAGE and Coomassie blue staining. Aliquoted proteins were frozen and stored at −80 °C.

### 2.2. DNA Substrate for Loading/Unloading Reaction

A 130-mer DNA substrate was prepared based on information provided in a previous report [11]. The annealed primer-template DNA was attached to streptavidin-coated magnetic beads (Dynabeads M-280 (Thermo Fisher Scientific, Waltham, MA, USA). Bead-attached DNA was re-suspended in 0.3 M KoAc Buffer H without magnesium acetate. The substrate had a TALE-binding sequence on the opposite side of biotinylation. For the reaction, 0.5 pmole of the DNA substrate was pre-incubated with a 50 nM TALE in a 40 µL reaction mixture (25 mM HEPES [pH 7.5], 300 mM KoAc, 1 mM EDTA, 1 mM EGTA, 2.5 mM magnesium acetate, 10% glycerol, 1 mM DTT, 1 mM ATP, 0.02 % NP40) at 37 °C for 30 min in order to prevent PCNA diffusion.

### 2.3. PCNA-Loading and Unloading Reaction

A standard 2× loading reaction buffer (50 mM HEPES [pH 7.5]), 24 mM magnesium acetate, 0.2 mM zinc acetate, 2 mM dithiothreitol [DTT], 40 mM phosphocreatine, 12 mM ATP, 0.04% NP40, 20% glycerol, 0.8 mg/mL bovine serum albumin, and 2× Complete Protease Inhibitor Cocktail (Merck, Darmstadt, Germany) and a protein dilution buffer (25 mM HEPES [pH 7.5], 300 mM KoAc, 1 mM EDTA, 1 mM EGTA, 2.5 mM magnesium acetate, 10% glycerol, 1 mM DTT, 1 mM ATP, and 0.02% NP40) was prepared. Initially, 20 μL of 2× loading reaction buffer was mixed with 20 μL of protein mix that contained RFC (12.5 nM or indicated amount) and PCNA (125 nM). The PCNA-loading reaction mixture was added to bead-conjugated TALE-bound DNA substrate. The reaction mixture was incubated in a Thermomixer (Eppendorf, Hamburg, Germany) for 30 min at 37 °C and 1200 rpm. After the loading reaction, the remaining RFC and unbound PCNA were removed by washing the beads once with 0.3 M KCl, twice with 0.5 M KCl, and once again with 0.3M KCl Buffer H.

For the PCNA unloading reaction, 20 μL of 2× loading reaction buffer was mixed with 20 μL of the protein mix containing various amounts of ATAD5-RLC. The unloading reaction mixture was added to the collected PCNA-loaded DNA beads. The unloading reaction mixture was incubated in a Thermomixer for 15 min at 37 °C. Afterward, the reaction beads were washed with 0.3M KCl Buffer H three times. Finally, DNA was re-suspended in 30 μL of digestion buffer (50 mM Tris-HCl [pH 7.5], 1 mM DTT, 4 mM magnesium chloride) containing 1 unit of DNase I (Promega, Madison, WI, USA). DNA was digested for 10 min, beads were collected using a magnet, and supernatants were taken to analyze PCNA level on DNA by immunoblotting.

### 2.4. Antibodies

The following antibodies were used: anti-PCNA (Rabbit), anti-LaminB1 antibodies (Abcam, Cambridge, UK); anti-FLAG, anti-HA antibodies (Merck, Darmstadt, Germany); anti-PCNA (mouse), anti-RFC4 antibodies (Santa Cruz, Dallas, TX, USA); anti-Myc antibody (Merck, Darmstadt, Germany). The anti-human ATAD5 antibody was raised in rabbits using the N-terminal 1–297 amino acid fragment and was then affinity-purified [12].

### 2.5. Plasmids (or) DNA Constructs and siRNAs

Plasmids expressing wild-type ATAD5, and its mutants were cloned into a p3× FLAG-CMV10 expression vector (Merck, Darmstadt, Germany) or pcDNA™5/FRT/TO (Thermo Fisher Scientific, Waltham, MA, USA). All constructs were confirmed by sequencing. The plasmids used in this work are listed in Table S1. Site-directed mutagenesis was performed using the QuikChange site-directed mutagenesis kit (Agilent Technologies, Santa Clara, CA, USA) according to the manufacturer’s instructions to generate plasmid DNA for ATAD5 M1, M2, M3, M4, M5, P1, and P2 mutants. All constructs were confirmed by sequencing.

The siRNA against the 3′ untranslated region (UTR) of ATAD5 5′-GUAUAUUUCUCGAUGUACA-3′ (sense) and 5′-UGUACAUCGAGAAAUAUAC-3′ (antisense) were synthesized and obtained from Bioneer (Daejeon, Korea). Non-targeting control siRNA (AccuTarget Negative Control siRNA) was purchased from Bioneer (Daejeon, Korea).

### 2.6. Cell Culture

Human embryonic kidney (HEK) 293T cells (purchased from American Type Culture Collection (Manassas, VA, USA) and their derivative cell lines stably expressing ATAD5 protein were cultured in Dulbecco’s modified Eagle’s medium (Cytiva, Marlborough, MA, USA) supplemented with 10% fetal bovine serum (Cytiva, Marlborough, MA, USA) and 1% penicillin-streptomycin (Thermo Fisher Scientific, Waltham, MA, USA) at 37 °C under 5% CO_2_.

### 2.7. Transfections and RNA Interference

Transfections of plasmid DNA were performed using X-tremeGENE HP DNA transfection Reagent (Merck, Darmstadt, Germany). In the case of siRNAs, we used Lipofectamine RNAiMAX (Thermo Fisher Scientific, Waltham, MA, USA), according to the manufacturer’s instructions. Transfected cells were harvested 48 h after transfection. To deplete only endogenous ATAD5, siRNA-targeting 3′UTR of ATAD5 was transfected 6 h after transfecting plasmids expressing wild-type or mutant ATAD5 protein.

### 2.8. Chromatin Fractionation

Cells were lysed with buffer A (100 mM NaCl, 300 mM sucrose, 3 mM MgCl_2_, 10 mM PIPES (pH 6.8), 1 mM EGTA, and 0.2% Triton X100, containing PhosSTOP (Merck, Darmstadt, Germany) and Complete Protease Inhibitor Cocktail (Merck, Darmstadt, Germany) for 8 min on ice. Lysates were centrifuged at 5000× *g* at 4 °C for 5 min to separate the chromatin-containing pellet. Supernatants were obtained as soluble fractions. The pellet was digested with 50 units of Benzonase (Enzynomics, Daejeon, Korea) for 40 min in RIPA buffer (50 mM Tris–HCl (pH 8.0), 150 mM NaCl, 5 mM EDTA, 1% Triton X-100, 0.1% SDS, 0.5% Na deoxycholate, 1 mM PMSF, 5 mM MgCl_2_, containing PhosSTOP (Merck, Darmstadt, Germany) and Complete Protease Inhibitor Cocktail (Merck, Darmstadt, Germany)) to extract chromatin-bound proteins. The chromatin-containing fractions were clarified by centrifugation (13,000× *g*, 4 °C) for 5 min to remove debris. The protein concentration was determined using the Bradford Assay (Bio-Rad, Hercules, CA, USA), and the proteins were analyzed by immunoblotting.

### 2.9. Immunoprecipitation and Western Blot

Whole-cell lysates were prepared with buffer X (100 mM Tris–HCl (pH 8.5), 250 mM NaCl, 1 mM EDTA and 1% Nonidet P-40, 5 mM MgCl_2_) containing PhosSTOP (Merck, Darmstadt, Germany), Complete Protease Inhibitor Cocktail (Merck, Darmstadt, Germany), and 500 units of Benzonase for 40 min at 4 °C. Lysates were cleared by centrifugation (13,000× *g*, 4 °C, 5 min). FLAG-tagged proteins were incubated with anti-FLAG M2 agarose affinity beads (Merck, Darmstadt, Germany) for 1 h at 4 °C. The beads were washed three times using buffer X, and the bead-bound proteins were eluted with buffer X containing 0.15 mg/mL 3× FLAG peptide. Co-immunoprecipitated proteins were analyzed by immunoblotting. The proteins were detected using the ChemiDoc MP imaging system (Bio-Rad, Hercules, CA, USA). The signal intensity of the bands was measured by ImageLab software version 5.2.1 (Bio-Rad, Hercules, CA, USA).

## 3. Results

### 3.1. ATAD5 (812–1844) Is a Minimal PCNA-Unloading Domain

**Figure 1:**
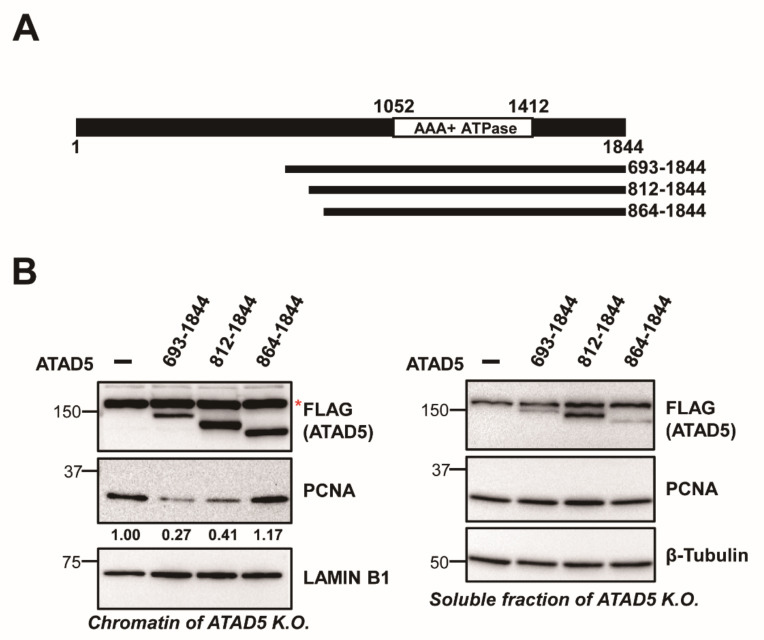
ATAD5 (812–1844) is a minimal PCNA unloading domain. (**A**) Diagram showing ATAD5 N-terminal deletion mutants. N-terminal 811 or 863 amino acids were deleted. (**B**) ATAD5 (812–1844) is a minimal PCNA unloading domain. Indicated ATAD5 variants were transiently transfected to ATAD5 knock-out 293T cells. After chromatin fractionation, the amount of chromatin-bound PCNA was analyzed by immunoblot. Chromatin-unbound soluble fractions were shown to examine total amount of each protein. Asterix indicates a non-specific band detected by anti-FLAG antibody in ATAD5 knock-out cells. ATAD5 (812–1844) reduced the amount of chromatin-bound PCNA, but ATAD5 (864–1844) did not. Number below the PCNA blot indicates relative amount of chromatin-bound PCNA.

Previously, we demonstrated that ATAD5 (693–1844) contains PCNA-unloading activity [11]. To further narrow down the PCNA-unloading domain, we prepared a series of N-terminal-truncated ATAD5 mutants (Figure 1A). We examined the PCNA-unloading activity of the ATAD5 variants by expressing them in ATAD5 knock-out 293T cells (Figure 1B) [18]. Knock-out of ATAD5 resulted in an increased amount of chromatin-bound PCNA (Figure S1A). The expression of ATAD5 (693–1844) reduced the amount of chromatin-bound PCNA as previously reported [11]. ATAD5 (812–1844) also reduced the levels of chromatin-bound PCNA. However, ATAD5 (864–1844) was unable to reduce the amount of chromatin-bound PCNA. Therefore, ATAD5 (812–1844) was found to be a ULD. These results suggest that the region of ATAD5 encompassing the 812nd and 863rd amino acids is important for the PCNA-unloading activity.

**Figure 2:**
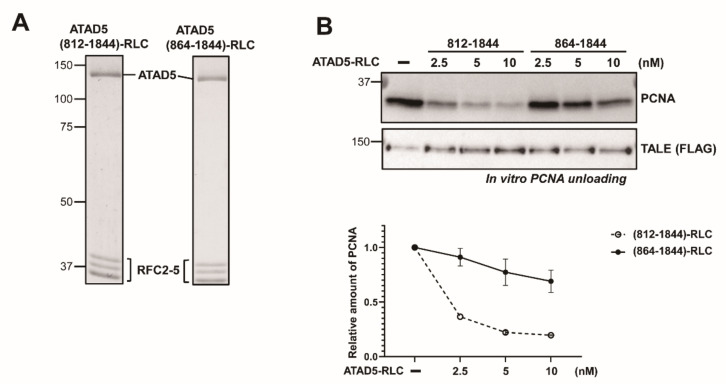
ATAD5 (812–1844) unloads PCNA in vitro. (**A**) Coomassie stained SDS-PAGE of purified ATAD5 (812–1844) and ATAD5 (864–1844). (**B**) ATAD5 (812–1844)-RLC unloads PCNA in vitro. *Top*—in vitro PCNA unloading assay was performed with ATAD5 (812–1844)-RLC and ATAD5 (864–1844)-RLC. ATAD5 (812–1844)-RLC efficiently released PCNA from DNA, but ATAD5 (864–1844)-RLC did not. The amount of TALE protein in each reaction was examined as a control (see methods). *Bottom*—relative PCNA amount on DNA was quantified. Error bars indicate standard deviation (*n* = 2).

Next, we biochemically examined whether the ATAD5 (812–863) region was crucial for PCNA unloading. We purified ATAD5 (812–1844) and ATAD5 (864–1844), each complexed with RFC2-5 (Figure 2A). Both of the ATAD5 variants robustly formed pentameric complexes. Therefore, ATAD5 (812–863) is not essential for RFC2-5 binding. However, ATAD5 (864–1844)-RLC was defective in PCNA unloading in vitro as in the cells (Figure 2B). To measure the PCNA-unloading activity, PCNA was first loaded onto the gapped DNA by RFC. After washing, purified ATAD5 (812–1844)-RLC or ATAD5 (864–1844)-RLC was treated to DNA-loaded PCNA. ATAD5 (812–1844) efficiently released PCNA from DNA, but ATAD5 (864–1844) did not. These results suggest that the ATAD5 (812–864) region is required for PCNA unloading. We designated ATAD5 (812–864) as the unloading regulatory motif (URM).

### 3.2. ATAD5 URM Is Required for PCNA Ring Opening

**Figure 3:**
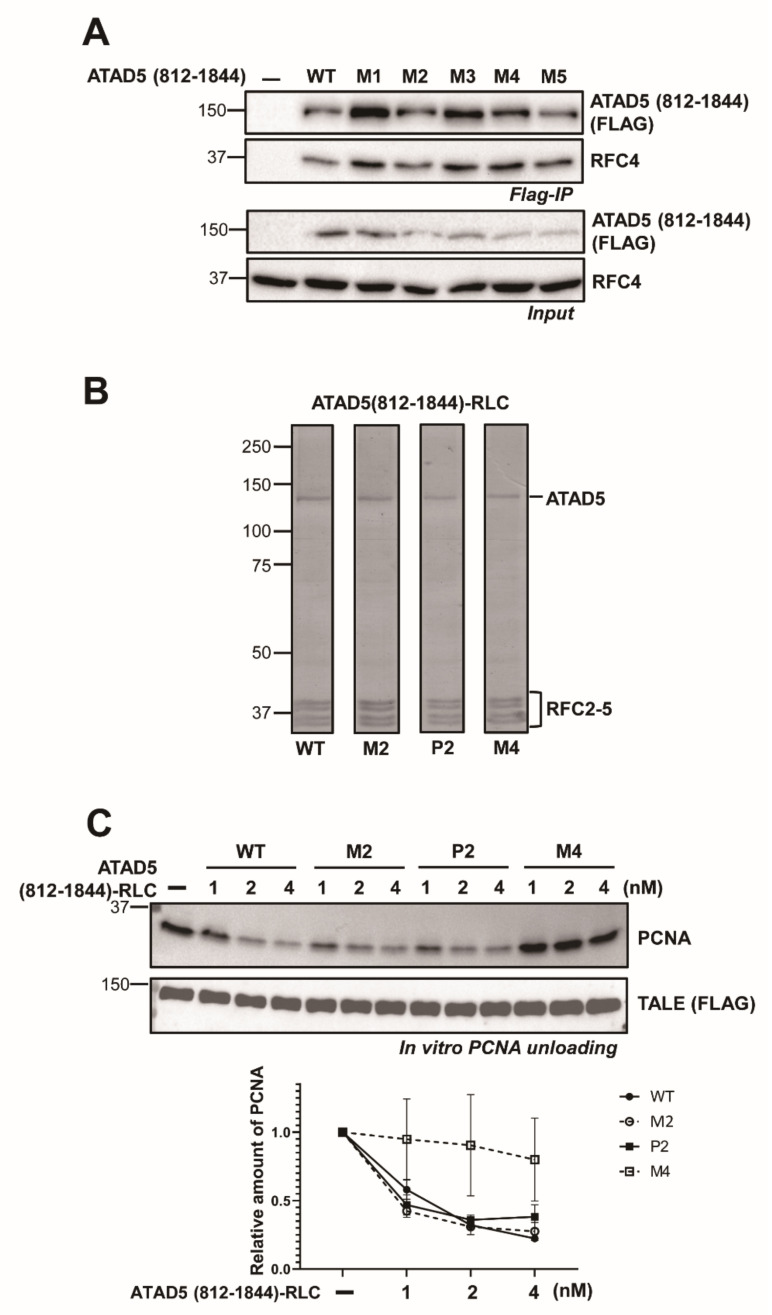
ATAD5 URM is important for efficient PCNA unloading. (**A**) RFC2-5 binding was not affected by URM mutations. URM mutants were transiently expressed in 293T cells. To monitor the RFC2-5 binding, FLAG-immunoprecipitation was performed and co-isolated RFC4 was analyzed by immunoblot. The amount of co-purified RFC4 was not changed by URM mutations. (**B**) Coomassie blue stained SDS-PAGE of URM mutant ATAD5 (812–1844)-RLC. URM mutants formed a pentameric complex with RFC2-5. (**C**) URM mutants are defective in PCNA unloading in vitro. *Top*—in vitro PCNA unloading assay was performed with URM mutants. Compared to wild type, URM mutants showed defects in releasing PCNA from DNA. *Bottom*—quantification of the relative PCNA amount on DNA. Error bars indicate standard deviation (*n* = 2).

The primary sequences of the URM are well conserved among species (Figure S2A). To understand the role of the URM in the PCNA-unloading process, we introduced several mutations to the conserved motifs in the URM. There are two SQ motifs, ^817^SQ^818^ and ^824^SQ^825^ in the N-terminus of the URM. These motifs are putative target sites for PI3 kinases. The phosphorylation of these two motifs can be found in phospho-protein databases [20]. We prepared two phospho-mimetic mutants, P1 (S817D) and P2 (S817D, S824D). On the other end, we generated two phospho-dead mutations, M1 (^815^SSQD^818^ to GGAA) and M2 (M1 plus ^824^SQD^826^ AAA). In the cases of M3 (^834^KRDF^837^ AAAA), M4 (^847^KRQ^849^ AAA) and M5 (^872^HVQQ^875^ AAAA), the clustered charged amino acids were mutated to alanine. M5 is located immediately downstream of the URM. All mutations were introduced into ATAD5 (812–1844). We expressed the mutant proteins in ATAD5-KO cells to examine their PCNA-unloading activity (Figure S2B). Compared to the wild-type, all mutants were at least partially defective in reducing the amount of chromatin-bound PCNA. In particular, the M5 mutation was severely defective in reducing PCNA levels on chromatin. To test whether mutant ATAD5 could form an intact RLC complex, we examined the interaction between the URM variants and endogenous RFC4 (Figure 3A). FLAG-immunoprecipitation was performed after transiently expressing the URM variant ATAD5, and co-purified RFC4 was detected. None of the URM mutants showed defects in RFC4 binding.

Next, we purified ATAD5 (812–1844)-RLCs with mutations in URM and analyzed their PCNA-unloading activity in vitro (Figure 3B,C). The P2, M2 and M4 mutants were prepared. The mutants efficiently formed pentameric complexes with RFC2-5. ATAD5 (812–1844) M5 mutant was not stably expressed in insect cells. Therefore, we could not obtain the intact M5 mutant complex for the in vitro assay (data not shown). We found that the P2 mutant was partially defective and the M4 mutant was severely defective in PCNA unloading. The M4 mutation delays PCNA release from DNA. These results suggest that the URM functions in the PCNA-unloading activity of ATAD5-RLC.

### 3.3. ATAD5 URM Contributes to the Opening of PCNA Ring

**Figure 4:**
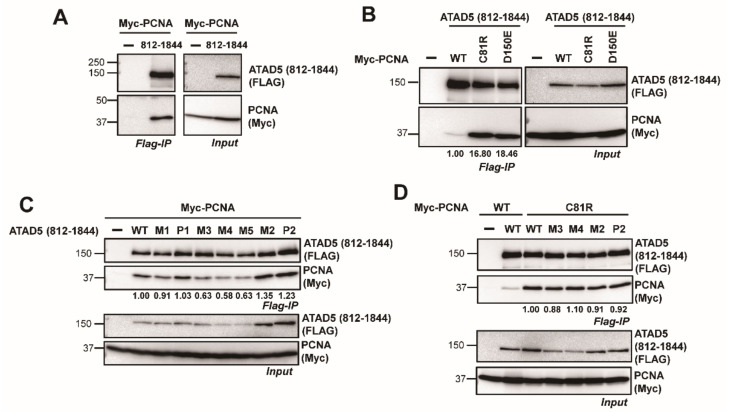
ATAD5 (812–1844) preferentially binds to the open form of PCNA. (**A**) ATAD5 (812–1844) binds to PCNA. FLAG-ATAD5 (812–1844) and Myc-PCNA were transiently co-expressed in 293T cells. FLAG-immunoprecipitation was performed and the co-isolation of Myc-PCNA was analyzed by immunoblot. (**B**) Open-liable PCNA more preferentially binds to ATAD5 (812–1844) compared to wild-type PCNA. Indicated PCNA variants were transiently co-expressed with ATAD5 (812–1844) and FLAG-immunoprecipitation was performed. Co-precipitated PCNA was analyzed by immunoblot. PCNA (C81R) and PCNA (D150E) were more enriched with ATAD5 (812–1844) compared to wild-type PCNA. (**C**) M3, M4 and M5 mutations in URM weakens PCNA binding. Indicated URM mutants were transiently co-expressed with Myc-PCNA, and FLAG immunoprecipitation was performed. Co-isolation of Myc-PCNA was analyzed by immunoblot. The amount of co-precipitated Myc-PCNA was reduced with M3, M4 and M5 mutants. Number below the IP-blot indicates relative amount of co-immunoprecipitated PCNA normalized with the amount of pull-downed bait. (**D**) URM mutants do not affect ATAD5 binding to open-liable PCNA. Indicated URM mutants were transiently co-expressed with Myc-PCNA (C81R), and FLAG immunoprecipitation was performed. Immunoblot results showed that PCNA (C81R) similarly bound to wild-type and mutant ATAD5 (812–1844). Number below the IP-blot indicates relative amount of co-purified PCNA (C81R) normalized with the amount of pull-downed bait.

During PCNA loading by RFC, PCNA first interacts with RFC1 and multiple interactions between PCNA and RFC subunits occur during the opening of the PCNA ring [21]. It is possible that the ATAD5-PCNA interaction dynamically changes during the PCNA-unloading process. We previously demonstrated that PCNA is released from DNA in the form of a PCNA-ATAD5-RLC intermediate [11]. ATAD5-RLC might bind more stably to the open PCNA-ring than the closed one. We detected an interaction between ATAD5 ULD and PCNA using immunoprecipitation (Figure 4A). To examine whether ATAD5 ULD preferentially binds to open PCNA, we adapted the open-liable PCNA mutations, C81R and D150E [22] Both mutations are located at the interface between subunits and are known to be easily open. The yeast PCNA (C81R) mutant did not stably form homotrimers [23]. If ATAD5 preferentially binds to open PCNA, the ATAD5-PCNA interaction can be strengthened by the C81R or D150E mutations. First, we tested whether the C81R mutation in human PCNA affects its stability on DNA. We purified PCNA (C81R) and examined its loading onto DNA using RFC in vitro (Figure S3A). PCNA (C81R) failed to be loaded stably onto DNA (Figure S3B). ATP hydrolysis is important for the separation of PCNA from RFC after loading. Therefore, RFC-PCNA accumulates on DNA in the presence of ATPγS, a non-hydrolysable ATP analogue [11]. PCNA (C81R) did not accumulate on DNA in the presence of ATPγS, unlike wild-type PCNA (Figure S3C). These results biochemically suggest that PCNA (C81R) is unstable on DNA, because the C81R mutation de-stabilizes the interfaces between PCNA subunits. Interestingly. ATAD5 (812–1844) was more strongly bound to PCNA (C81R) and PCNA (D150E) than to wild-type PCNA (Figure 4B). These results imply that ATAD5 preferentially binds to the open form of PCNA.

Next, we examined whether mutations in the URM affected the dynamic interactions between ATAD5 and PCNA (Figure 4C,D). Interestingly, M3, M4 and M5 mutations weakened binding to wild-type PCNA (Figure 4C). To examine whether URM is sufficient to interact with PCNA, we examined the interaction between PCNA and ATAD5 (693–984), which contains a URM region (Figure S3D). We found that ATAD5 (693–984) did not efficiently bind to PCNA. The M4 mutation did not affect the weak interaction between PCNA and ATAD5 (693–984). These results imply that although URM is important for PCNA-ring opening, the whole architecture of ATAD5 C-terminal domain is required for the URM function. Compared to wild-type PCNA, URM mutations did not significantly affect binding to PCNA (C81R) (Figure 4D). To address the role of URM in binding to open-liable PCNA, we tested the interaction between ATAD5 (864–1844) and open-liable PCNA (C81R) (Figure S3E). Consistent with PCNA unloading (Figure 2B), wild-type PCNA bound less strongly to ATAD5 (864–1844) compared to ATAD5 (812–1844). However, the PCNA (C81R) mutation significantly enhanced the binding to ATAD5 (864–1844). These results support our hypothesis that ATAD5 URM is important for PCNA-ring opening and imply that the URM mutation does not strongly affect ATAD5 binding to open PCNA. It is possible that URM functions in the generation of the ATAD5-bindable open form of PCNA.

### 3.4. SUMO2 Fusion to PCNA Does not Enhance Unloading

**Figure 5:**
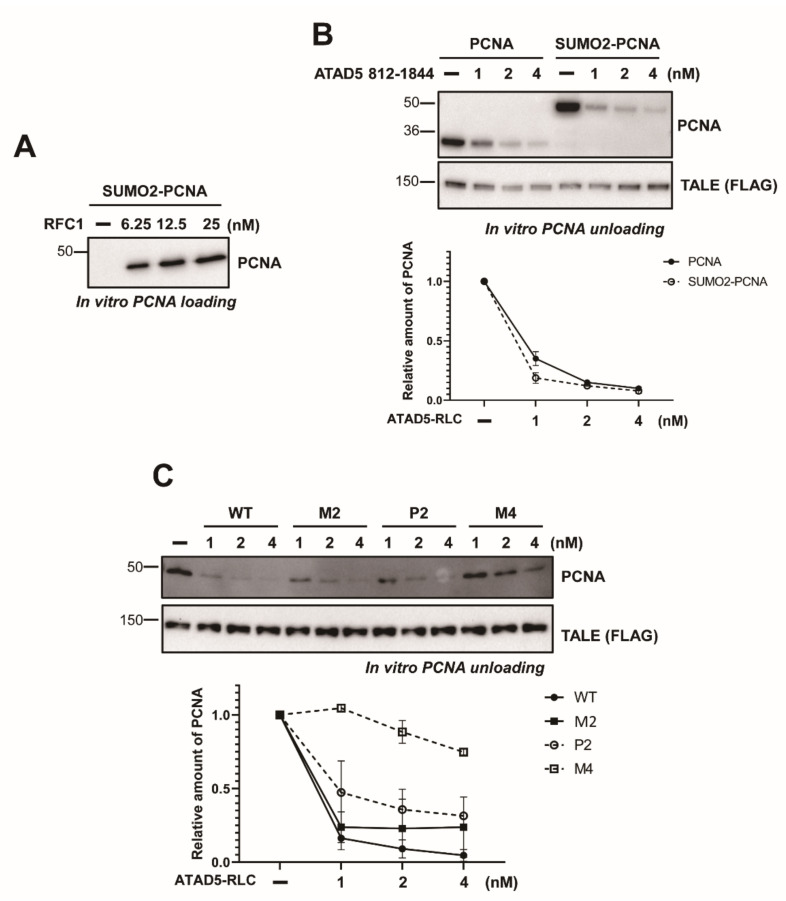
SUMO2 fusion does not affect PCNA unloading. (**A**) SUMO2-PCNA is efficiently loaded to DNA by RFC in vitro. PCNA loading assay was performed with SUMO2-PCNA. (**B**) ATAD5 (812–1844)-RLC can unload PCNA and SUMO2-PCNA in similar efficiency. *Top*—PCNA or SUMO2-PCNA were subjected to PCNA-unloading reaction with wild-type ATAD5 (811-1844)-RLC. There is no significant difference in unloading efficiency between PCNA and SUMO2-PCNA unloading. *Bottom*—Quantification of the relative PCNA amount on DNA. Error bars indicate standard deviation (*n* = 2). (**C**) URM mutants were defective in SUMO2-PCNA unloading in vitro. *Top—*PCNA unloading assay was performed with indicated ATAD5-RLC variants and SUMO2-PCNA. ATAD5 URM mutants showed similar defects in SUMO2-PCNA unloading and unmodified PCNA unloading (see Figure 3C). *Bottom—*quantification of the relative SUMO2-PCNA amount on DNA. Error bars indicate standard deviation (*n* = 2).

It has been reported that chromatin-accumulated PCNA is SUMOylated (SUMO-PCNA) in budding yeast and SUMO-PCNA is a preferred target of Elg1-RLC, a yeast homologue of ATAD5-RLC [24,25]. Although the role of SUMO-PCNA has not been well characterized in human cells, it is possible that PCNA SUMOylation affects the PCNA-unloading process. We examined whether SUMO2 fusion with PCNA affected PCNA unloading in vitro. N-terminal SUMO2-fused PCNA (SUMO2-PCNA) was purified and used for in vitro experiments (Figure S4A). SUMO2 fusion did not affect the PCNA loading by RFC (Figure 5A). In addition, SUMO2 fusion did not enhance the unloading of PCNA by ATAD5 (812–1844)-RLC (Figure 5B). URM mutations reduced SUMO2-PCNA unloading to a degree similar to that in unmodified PCNA (Figure 5C). These results suggest that SUMO2 fusion to PCNA does not enhance the PCNA-unloading activity of ATAD5-RLC. Although SUMO2-fusion did not affect PCNA unloading, SUMO2-PCNA bound to the ATAD5 ULD more strongly than unmodified PCNA (Figure S4B). URM mutations reduced SUMO2-PCNA binding similarly to unmodified PCNA (Figure S4C). These results suggest that the enhanced interaction between the ATAD5 ULD and PCNA by SUMO2 fusion does not contribute directly to the unloading process. URM does not seem to play a role in the recognition of the SUMO2 motif.

### 3.5. The ATAD5 C-Terminus Is Important for Stable RFC2-5 Binding

**Figure 6:**
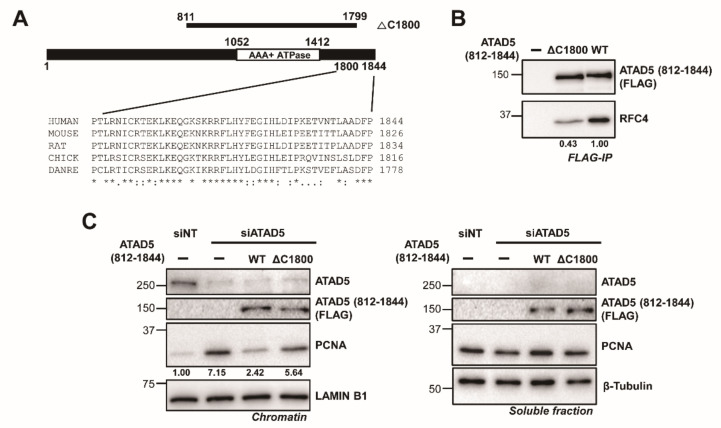
ATAD5 C-terminus is required for efficient PCNA unloading. (**A**) ATAD5 C-terminus is conserved among species. Primary sequences of ATAD5 C-terminus from different species are aligned. (**B**) ATAD5 C-terminus is required for stable RFC2-5 binding. ATAD5 (812–1844) or ATAD5 (812–1799) were transiently expressed and FLAG-immunoprecipitation was performed. The amount of co-precipitated RFC4 was reduced with ATAD5 (812–1799). (**C**) ATAD5- C-terminus is essential for PCNA unloading. ATAD5 (812–1844) or ATAD5 (812–1799) were transiently expressed in ATAD5 depleted cells. After chromatin fractionation, the amount of chromatin-bound PCNA was monitored by immunoblot. Chromatin-unbound soluble fractions were shown to examine total amount of each protein. ATAD5 (812–1799) failed to reduce PCNA level on chromatin compared to ATAD5 (812–1844). Number below the PCNA blot indicates relative amount of chromatin-bound PCNA.

In addition to URM, the C-terminus of the ATAD5 ULD is well conserved (Figure 6A). The C-terminal domain of ATAD5, ATAD5 (1600–1844), is known to function as an RFC2-5 binding region and is referred to as the collar domain [11]. Previously, we showed that the ATAD5 (1600–1800) region is essential for RFC2-5 binding. However, the role of the ATAD5 C-terminus, ATAD5 (1800–1844), has not been identified, although the region is highly conserved. We first examined whether the C-terminus was required for RLC formation. ATAD5 (1800–1844) was deleted from the ULD and the interaction with RFC2-5 was analyzed by Immunoprecipitation (Figure 6B). The deletion of ATAD5 (1800–1844) did not eliminate but significantly reduced the co-immunoprecipitation of RFC4. This result suggests that ATAD5 (1800–1844) enhances the binding of RFC2-5 to ATAD5 for stable ATAD5-RLC formation.

Next, we examined the PCNA-unloading activity of ATAD5 (812–1799) in ATAD5-depleted cells (Figure 6C). The C-terminal-deletion mutant failed to reduce the amount of chromatin-bound PCNA. Therefore, the stable association of RFC2-5, which is mediated by the ATAD5 C-terminus, is crucial for PCNA unloading by ATAD5-RLC.

## 4. Discussion

In eukaryotic cells, PCNA unloading is mediated by a specialized clamp-loader complex, ATAD5-RLC [11,12]. A recent report revealed the potent PCNA-unloading activity of purified ATAD5-RLC, but the detailed PCNA-unloading process remains to be elucidated [11]. In this study, we determined the minimal domain of ATAD5, ULD, which is sufficient for PCNA-unloading activity. In addition, we found that the N-terminus and the C-terminus of ULD had distinct functions in PCNA unloading.

Recent studies on RFC-PCNA interactions during PCNA loading provide insight into how ATAD5-RLC opens PCNA during the unloading [21]. Similar to RFC, ATAD5 forms a ring-shaped pentameric complex with RFC2-5. According to our hypothesis, ATAD5 might initially recognize PCNA (Figure S5). The interaction between ATAD5-RLC and PCNA may be intensified during the opening of the PCNA ring. For this process, ATAD5 and RFC2-5 should be aligned correctly. We found that the deletion of the ULD C-terminus de-stabilized the interaction between ATAD5 and RFC2-5 (Figure 6). We hypothesize that the C-terminal deletion hindered the correct positioning of RFC2-5 in ATAD5-RLC during the PCNA-unloading process, resulting in an unloading defect.

The enhanced interaction between ATAD5-RLC and open-liable PCNA supports the idea that the interaction between ATAD5-RLC and PCNA intensifies as the PCNA ring opens (Figure 4). We hypothesize that PCNA (C81R) or PCNA (D150E) can be spontaneously opened and could generate more open-form PCNA with a higher affinity for ATAD5-RLC than closed PCNA. 

We found that the deletion of the upper region of the ATAD5 ULD substantially reduced the PCNA-unloading activity (Figure 1 and Figure 2). Mutations in the URM also reduced PCNA-unloading activity in vitro (Figure 3). Interestingly, M3, M4, and M5 mutations reduced PCNA binding (Figure 4). Because these mutations did not affect the binding to open-liable PCNA, URM was not essential for binding to open PCNA, which was generated at later stages of the unloading process. We hypothesize that URM might function in the early stages of PCNA unloading, either in initial PCNA recognition or in the transition from closed to open PCNA. During the transition to opening PCNA, the URM might have a role in modulating the interactions between PCNA and ATAD5-RLC subunits. In the case of open-liable PCNA, a URM defect could be bypassed by the spontaneous ring opening of PCNA. There are two SQ sites in URM (Figure S3A). Phospho-mimetic mutations at these sites did not significantly reduce PCNA-unloading activity (Figure 3C). This result implies that the phosphorylation itself may not modulate unloading activity. BET proteins bind upstream of the ULD and fine-tune PCNA unloading activity [19]. It is possible that unidentified phospho-specific binding proteins interact with URM after phosphorylation and regulate PCNA unloading.

Previously, our group showed that the ubiquitination of PCNA at K164 did not affect PCNA unloading by ATAD5-RLC [11]. In addition to ubiquitination, K164 is also known to be SUMOylated. SUMO1- or SUMO2- conjugation in PCNA has been previously reported [24,26]. Whether SUMOylation affects PCNA unloading by human ATAD5-RLC has not yet been investigated. We examined ATAD5-RLC mediated unloading of N-terminal SUMO2-fused PCNA in the current study. Our experiments have limitations in that SUMO2-fusion does not fully recapitulate PCNA SUMOylation. Nevertheless, our results showed that SUMO2-fusion does not affect PCNA unloading in vitro. This result suggests that SUMO2, on PCNA, does not enhance the PCNA-ATAD5 interaction during PCNA ring opening by the ATAD5 ULD. We found that the SUMO2-fusion enhanced binding to ATAD5 (812–1844). Because SUMO2-fusion did not affect PCNA unloading, we think that enhanced binding does not contribute to the PCNA-ATAD5 interactions required for PCNA unloading. The role of PCNA SUMOylation in ATAD5 functions should be investigated in future studies.

## Data Availability

Original gel or blot images are available in the Supplementary Materials.

## Supplementary Materials

The following supporting information can be downloaded at: https://www.mdpi.com/article/10.3390/cells11111832/s1, Figure S1: ATAD5 knock out resulted in increase of chromatin-bound PCNA; Figure S2: ATAD5 URM mutants are defective in PCNA unloading in cells; Figure S3: PCNA (C81R) is unstable on DNA in vitro; Figure S4: SUMO2 fusion to PCNA enhances ATAD5 binding, but did not affect the opening of PCNA ring by ATAD5-RLC; Figure S5: Proposed model for PCNA unloading process by ATAD5-RLC; Table S1: Plasmids used in this study.
