# Supplementary material for: Distinct Motifs in ATAD5 C-Terminal Domain Modulate PCNA Unloading Process
Source: Cells. 2022 Jun 3;11(11):1832. doi: 10.3390/cells11111832 (PMC9180478; doi:10.3390/cells11111832)

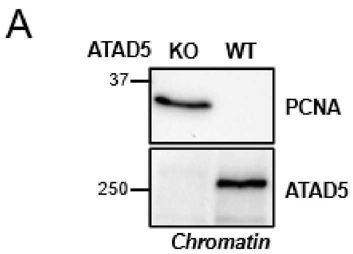


**Figure S1.** ATAD5 knock out resulted in increase of chromatin-bound PCNA. (**A**) PCNA is accumulated on chromatin in ATAD5 knock-out cells. Full length ATAD5 was re-expressed in ATAD5 knock-out cells to monitor the effect of ATAD5 deletion. Chromatin fractions were prepared from ATAD5 knock-out 293T cells (KO) and ATAD5 add back (WT). In the absence of ATAD5, PCNA was accumulated on chromatin.

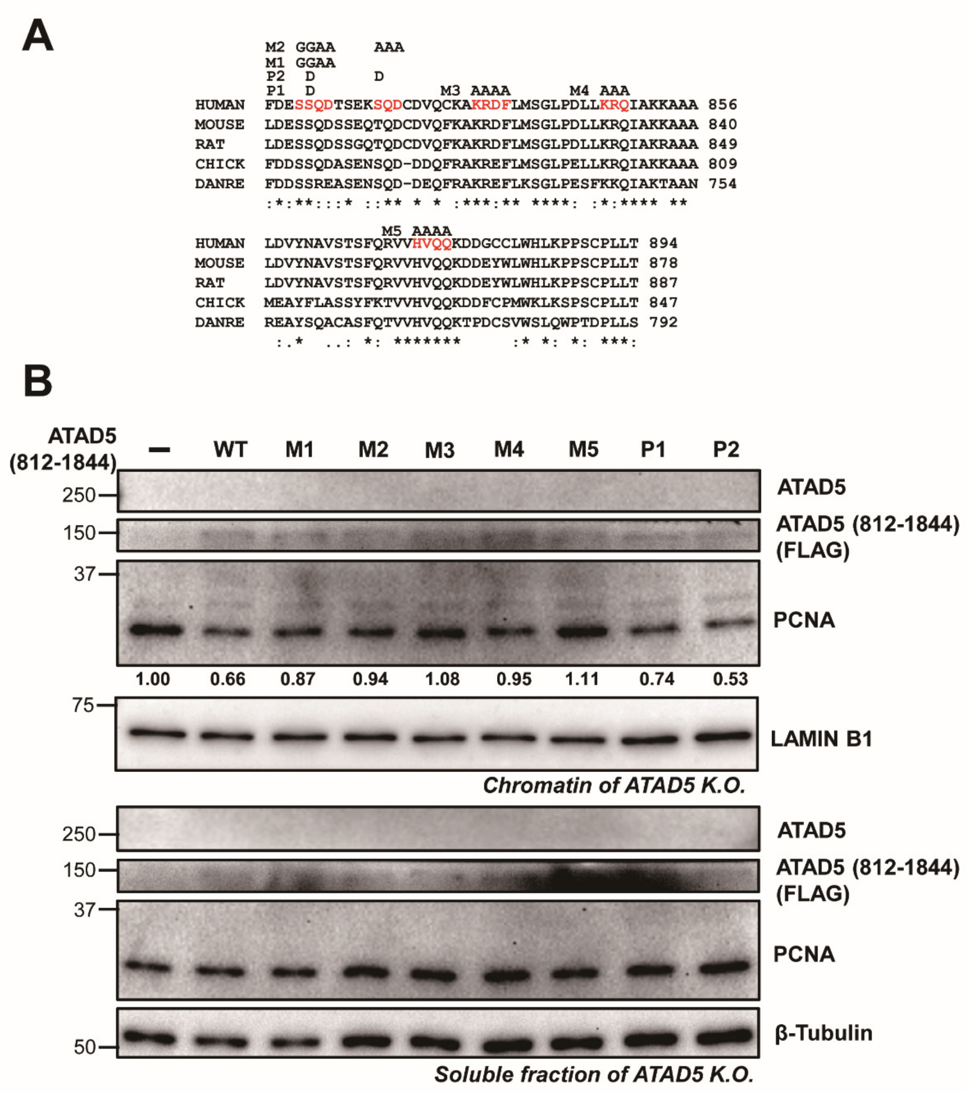


**Figure S2.** ATAD5 URM mutants are defective in PCNA unloading in cells. (**A**) Sequence comparison of ATAD5 URMs among different species. Positions of mutations analyzed in this study was indicated. (**B**) URM mutations reduce PCNA-unloading activity. Indicated URM mutants were transiently expressed in ATAD5-KO cells. After chromatin fractionation, the amount of chromatin-bound PCNA was analyzed by immunoblot. URM mutants did not fully reduce the PCNA amount on chromatin compared to wild-type. Number below the PCNA blot indicate relative amount of chromatin-bound PCNA.

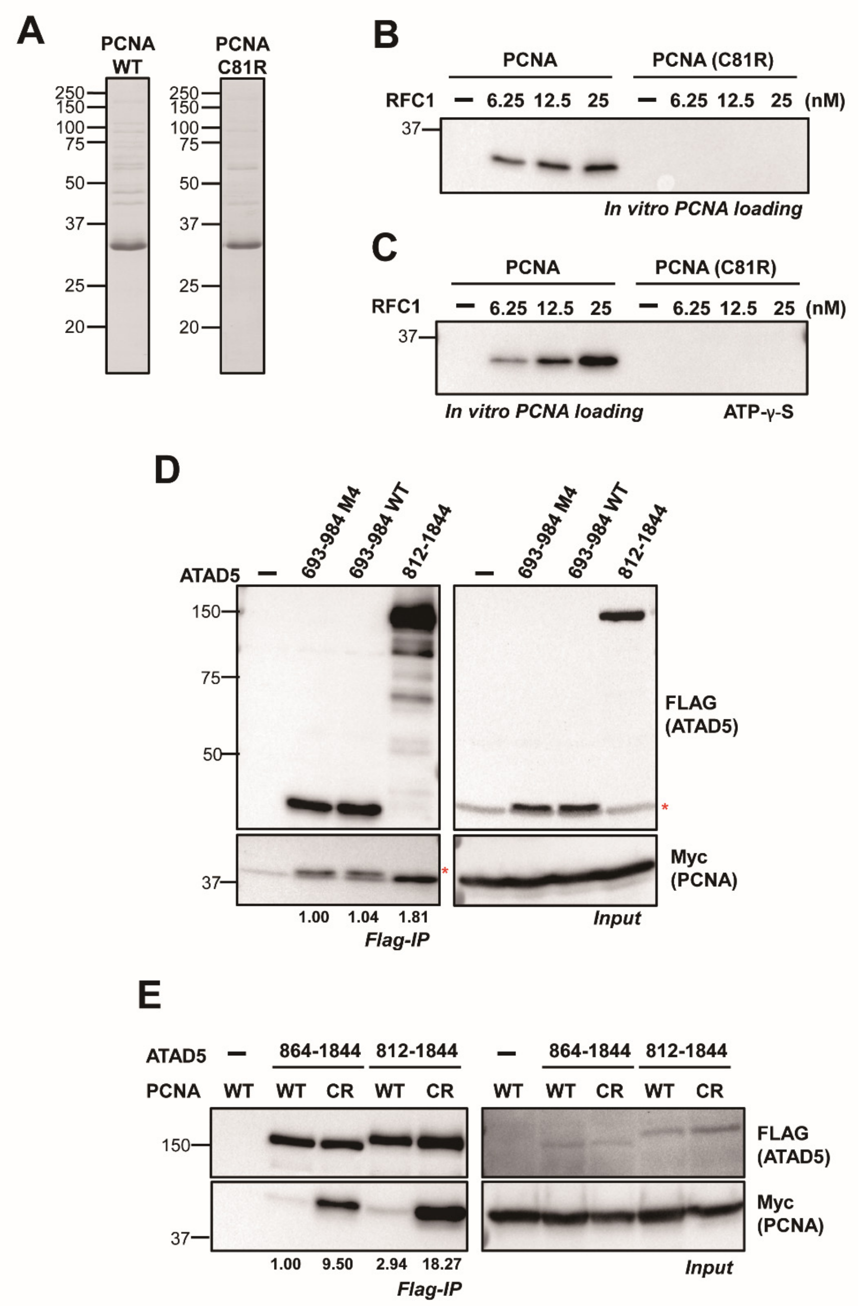


**Figure S3.** PCNA (C81R) is unstable on DNA in vitro. (**A**) Coomassie stained SDS-PAGE of purified PCNA and PCNA (C81R). (**B**) PCNA (C81R) is defective in DNA loading. PCNA-loading assay was performed with gapped DNA and RFC. The amount of DNA-loaded PCNA was analyzed by immunoblot. PCNA (C81R) was not retained on DNA after the loading reaction. (**C**) PCNA (C81R) is not accumulated on DNA in the presence of ATP-γ-S and RFC. PCNA-loading assay was performed in the presence of ATP-γ-S, a non-hydrolysable ATP analogue. Wild-type PCNA accumulated on DNA, but PCNA (C81R) did not. (D) ATAD5 (693-984) did not efficiently bind to PCNA. FLAG-tagged URM containing ATAD5 (693-984) wild-type or M4 mutant were transiently expressed in 293T cells and FLAG immunoprecipitation was performed. PCNA was not efficiently co-immunoprecipitated with ATAD5 (693-984) and M4 mutation did not affect weak PCNA co-immunoprecipitation. (E) ATAD5 (864-1844) binds to open-liable PCNA. PCNA (C81R) ,CR, was co-expressed with FLAG-tagged ATAD5 (812-1844) or ATAD5 (864-1844) and FLAG-immunoprecipitation was performed. C81R mutation in PCNA strengthen the interaction between PCNA and ATAD5 (864-1844).

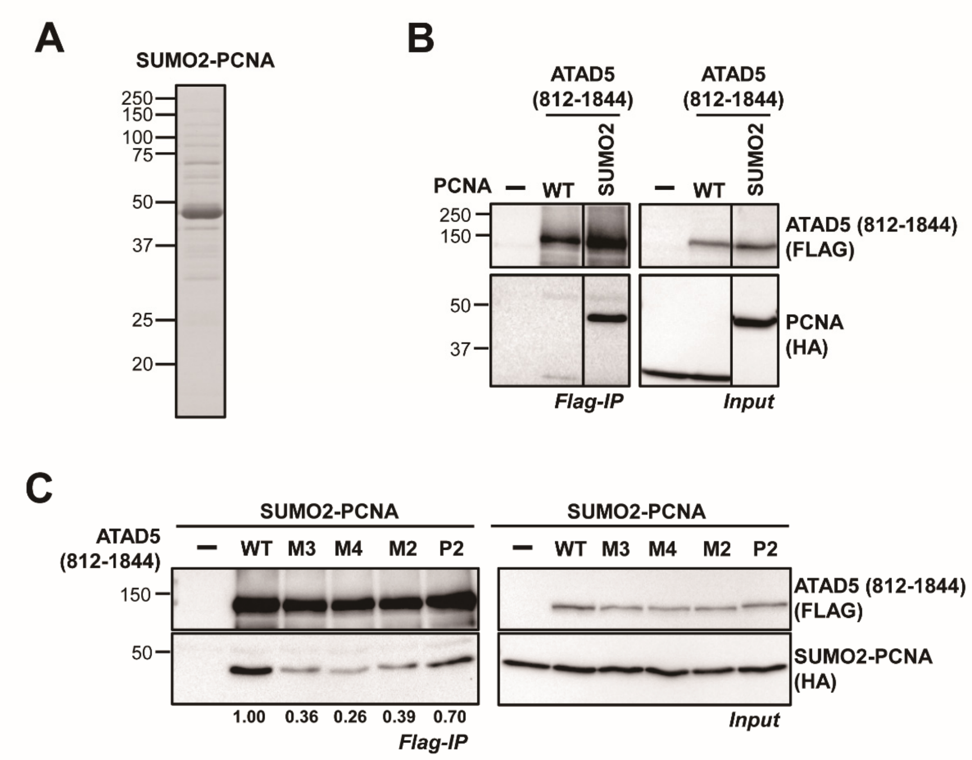


**Figure S4.** SUMO2 fusion to PCNA enhances ATAD5 binding, but did not affect the opening of PCNA ring by ATAD5-RLC. (**A**) Coomassie stained SDS-PAGE of purified SUMO2-fused PCNA. (**B**) SUMO2-fusion to PCNA enhances the interaction between ATAD5 (812-1844) and PCNA. Indicated HA-tagged PCNA variants were transiently co-expressed with ATAD5 (812-1844) and FLAG immunoprecipitation was performed. Immunoblot of precipitants showed that ATAD5 (812-1844) more strongly bound to SUMO2-fused PCNA. (**C**) URM mutants reduce the SUMO2-PCNA binding like unmodified PCNA. Indicated URM mutants of ATAD5 (812-1844) were transiently co-expressed with SUMO2-PCNA and FLAG-immunoprecipitation was performed. M2, M3 and M4 mutations reduced SUMO2-PCNA binding. Numbers under the IP-blot indicate relative amount of co-purified SUMO2-PCNA normalized with the amount of pull-downed bait.

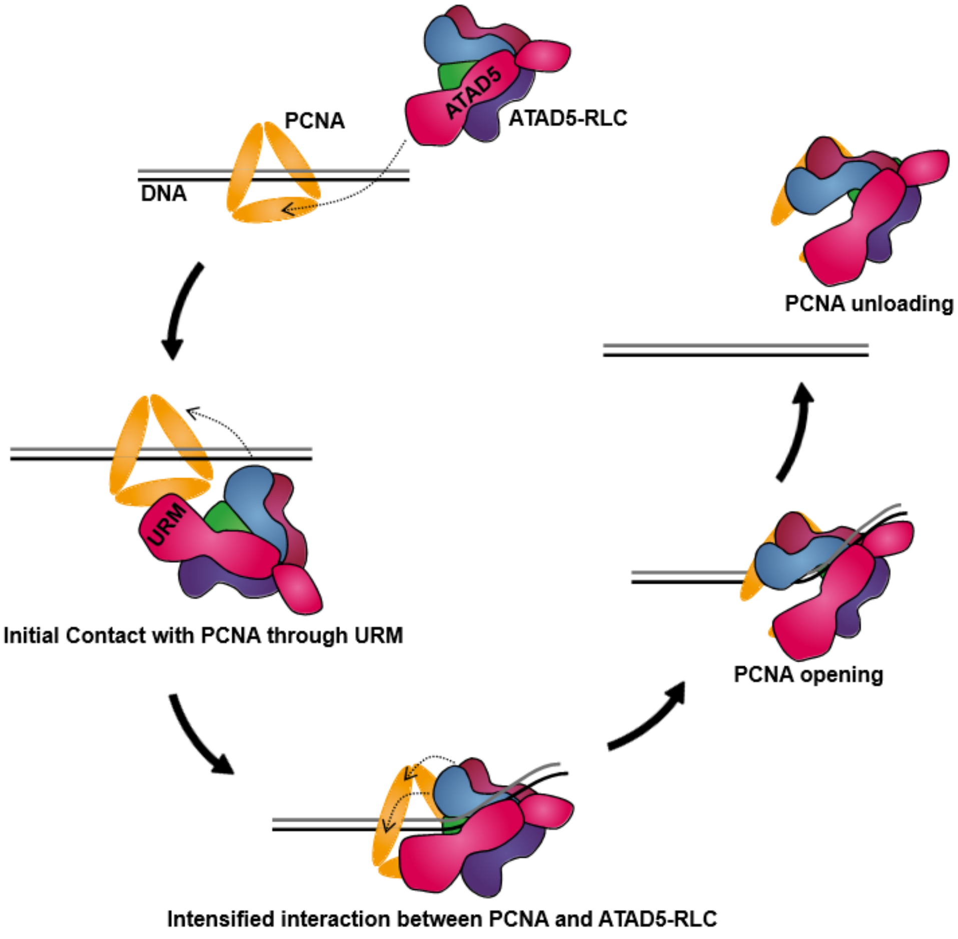


**Figure S5.** Proposed model for PCNA unloading process by ATAD5-RLC. ATAD5 initially recognizes PCNA. Next, binding between ATAD5-RLC and PCNA intensifies to open PCNA-ring. Finally, PCNA-ATAD5-RLC intermediate is released from DNA.

**Table S1.** Plasmids used in this study.

| Plasmids | Description | Source/ Reference |
|---|---|---|
| pMBS114 | pcDNA5/FRT/TO (ATAD5 (1-32+812-1844)-2xSTREPII-3xFLAG) | This study |
| pMBS115 | pcDNA5/FRT/TO (ATAD5 (1-32+864-1844)-2xSTREPII-3xFLAG) | This study |
| pMBS116 | pMBS114 + M1 | This study |
| pMBS117 | pMBS114 + M2 | This study |
| pMBS118 | | This study |
| pMBS119 | pMBS114 + M4 | This study |
| pMBS120 | pMBS114 + M5 | This study |
| pMBS121 | pMBS114 + P1 | This study |
| pMBS122 | pMBS114 + P2 | This study |
| pMBS123 | pcDNA5/FRT/TO (ATAD5 (1-32+812-1799)-2xSTREPII-3xFLAG) | This study |
| pMBS124 | pcDNA5/FRT/TO (3xMyc-PCNA) | This study |
| pMBS125 | pMBS124 + C81R | This study |
| pMBS126 | pMBS124 + D150E | This study |
| pMBS127 | pcDNA5/FRT/TO (3xHA-PCNA) | This study |
| pMBS128 | pcDNA5/FRT/TO (3xHA-SUMO2-PCNA) | This study |
| pMBS129 | pGEX6P3-HA-PCNA | This study |
| pMBS130 | pGEX6P3-HA-PCNA (C81R) | This study |
| pMBS131 | pGEX6P3-HA-SUMO2-PCNA | This study |

# Original Data

**Figure 1B**

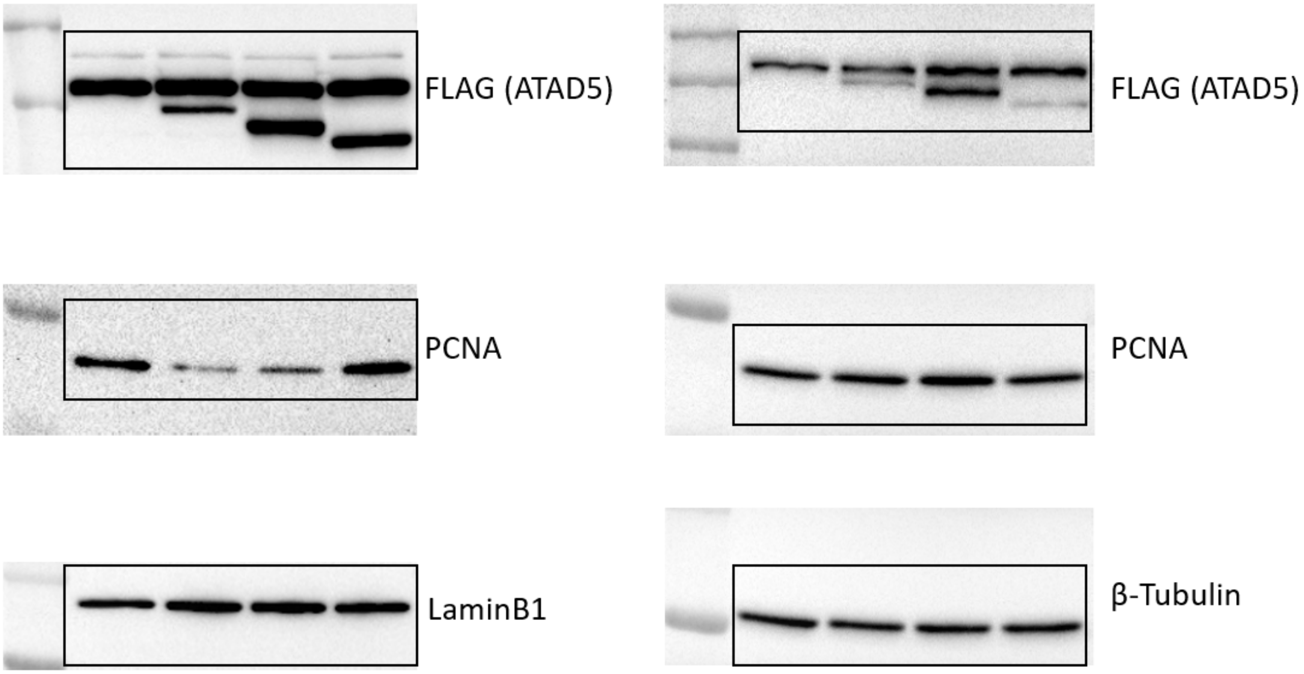

**Figure 1B_repeat**

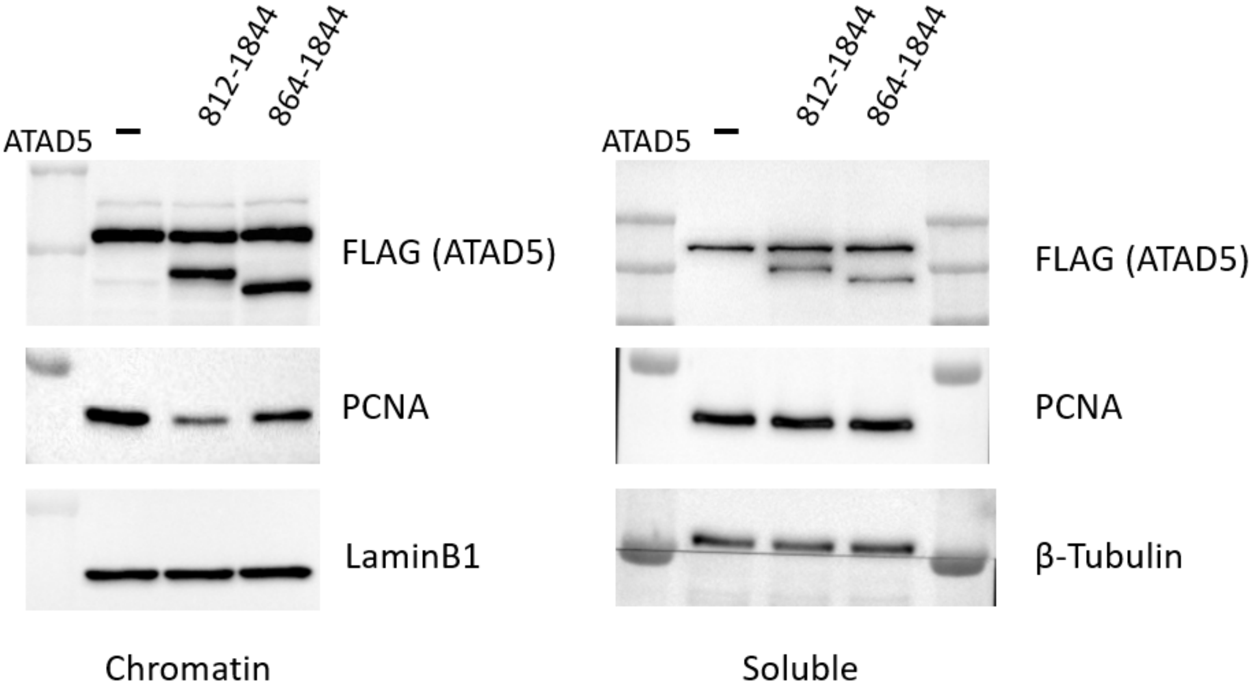

**Figure 2A**

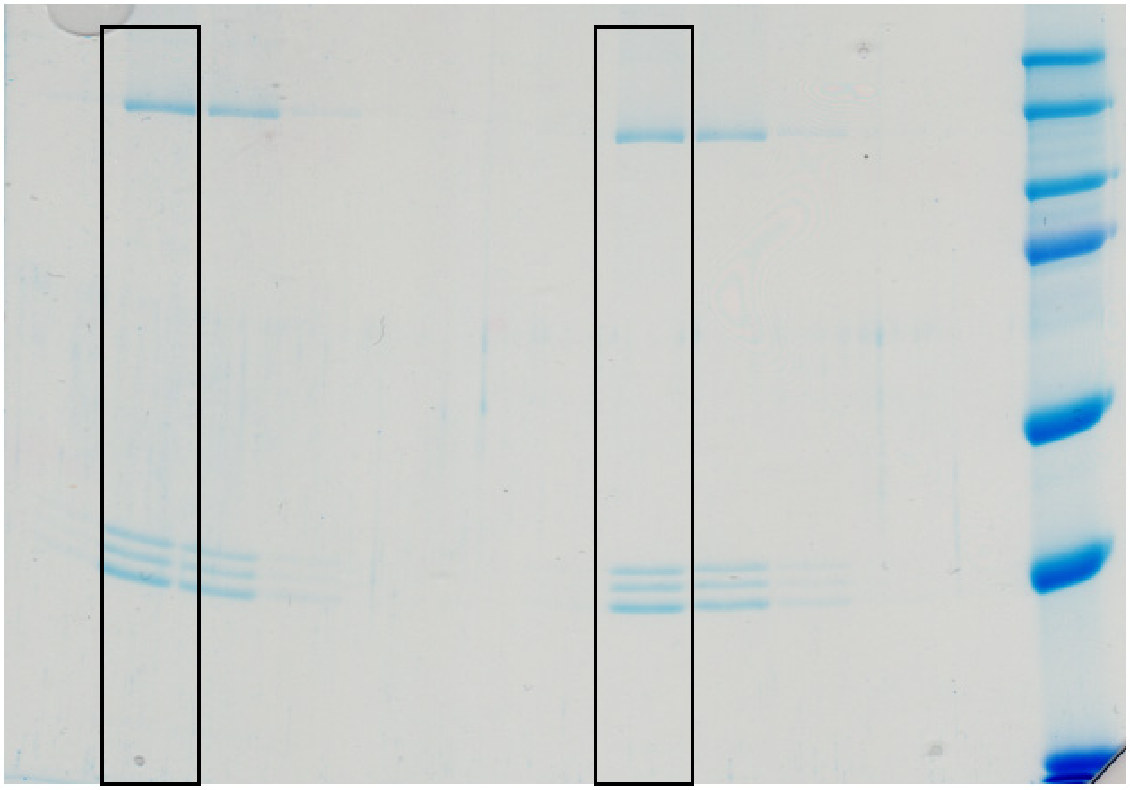

**Figure 2B**

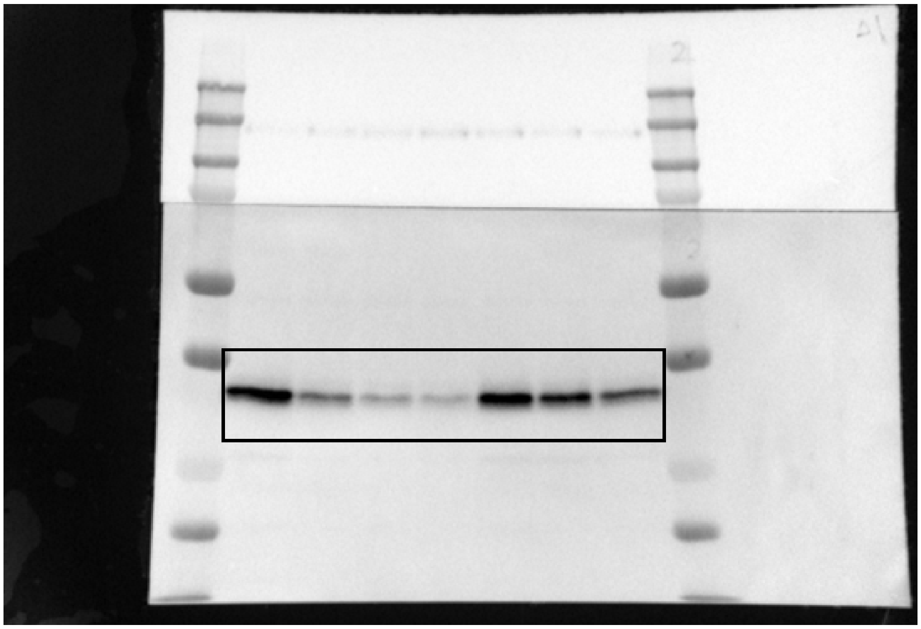

PCNA

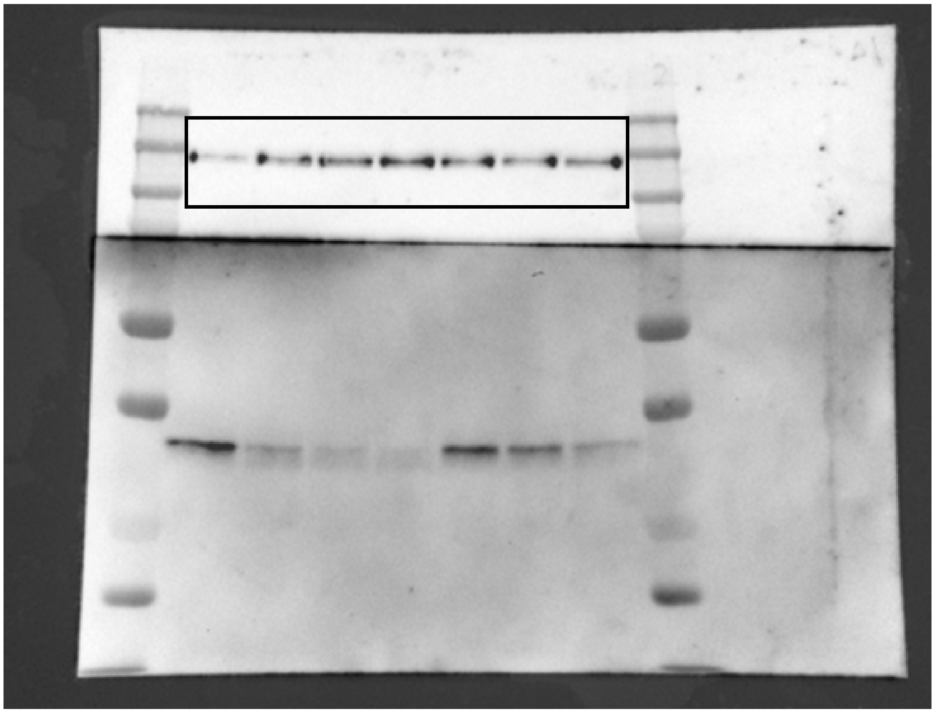

TALE (FLAG)

**Figure 2B_repeat**

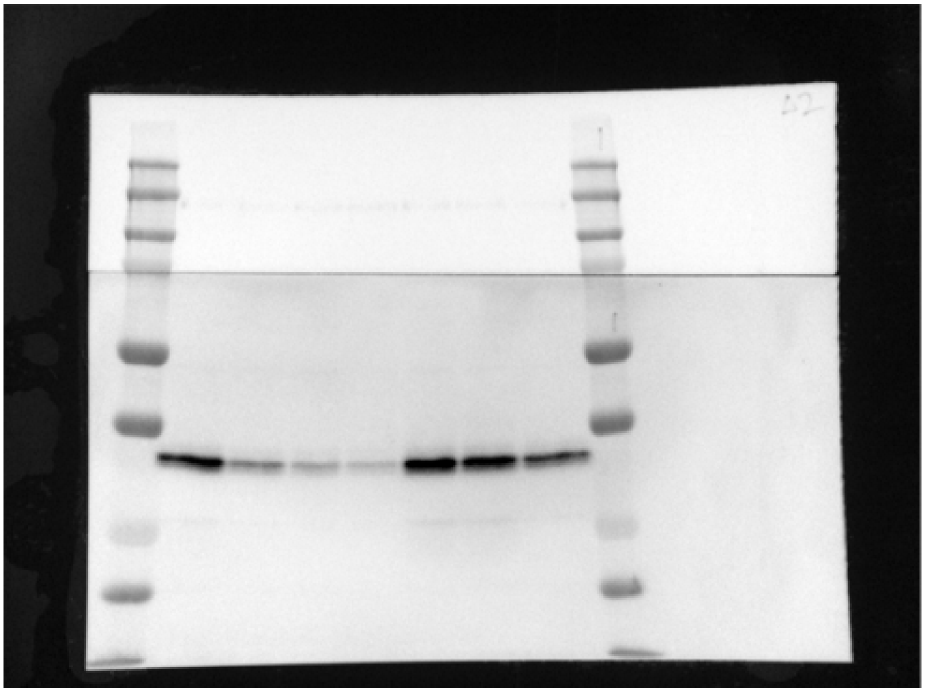

PCNA

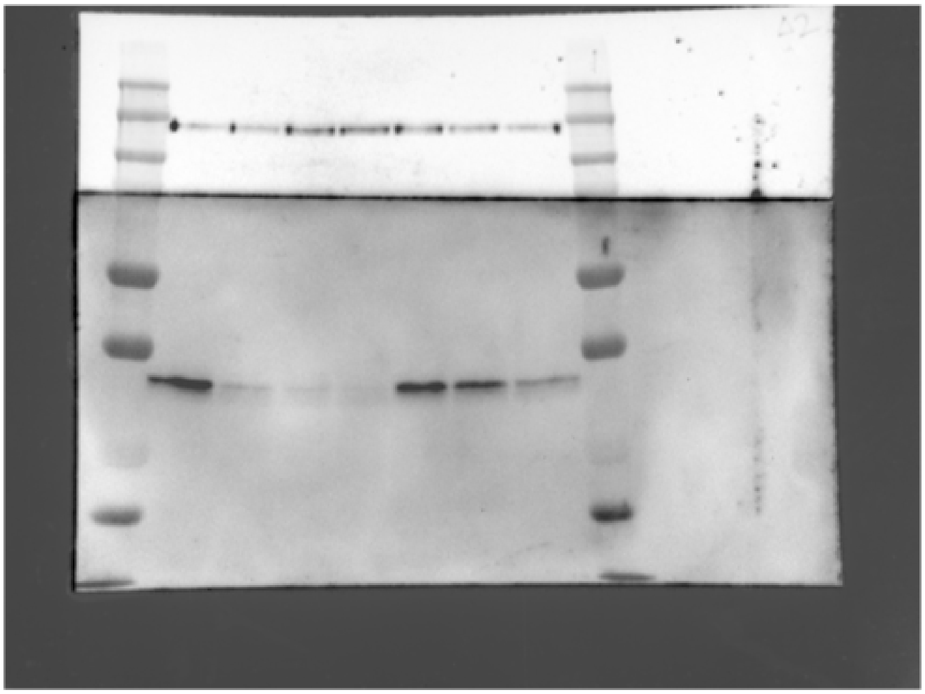

TALE (FLAG)

**Figure 3A**

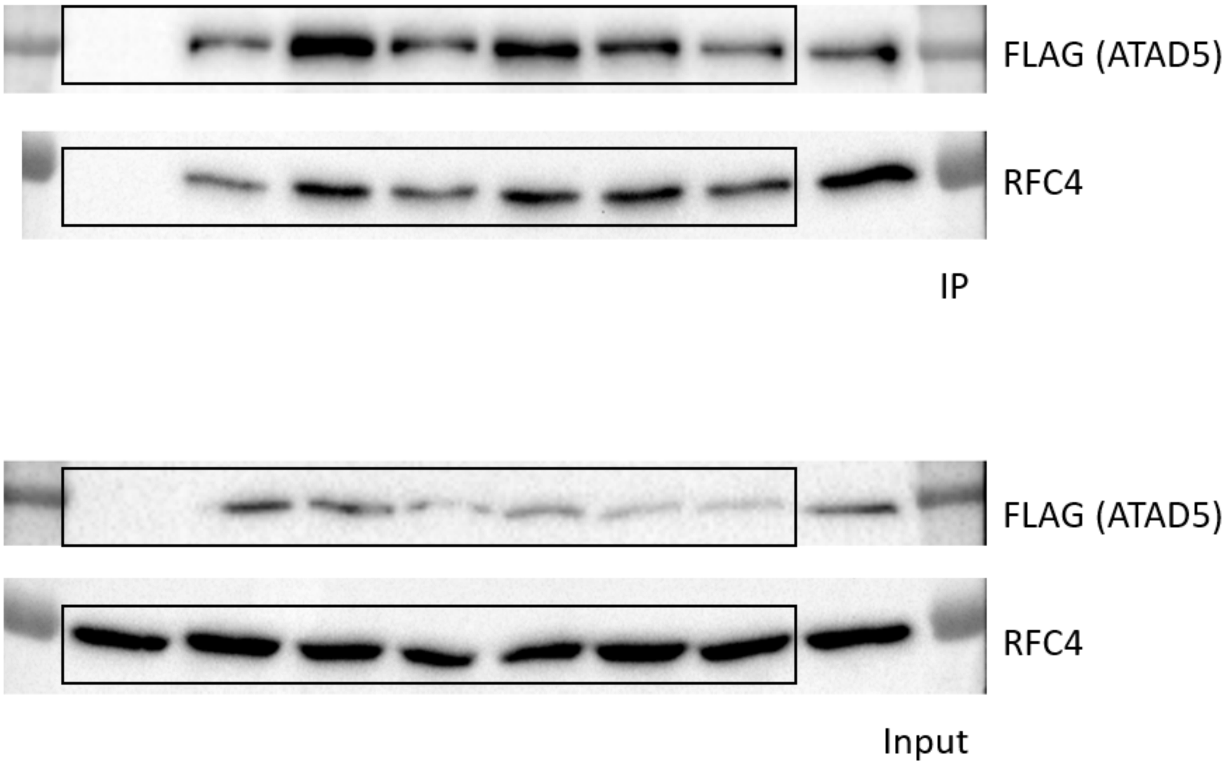

**Figure 3B**

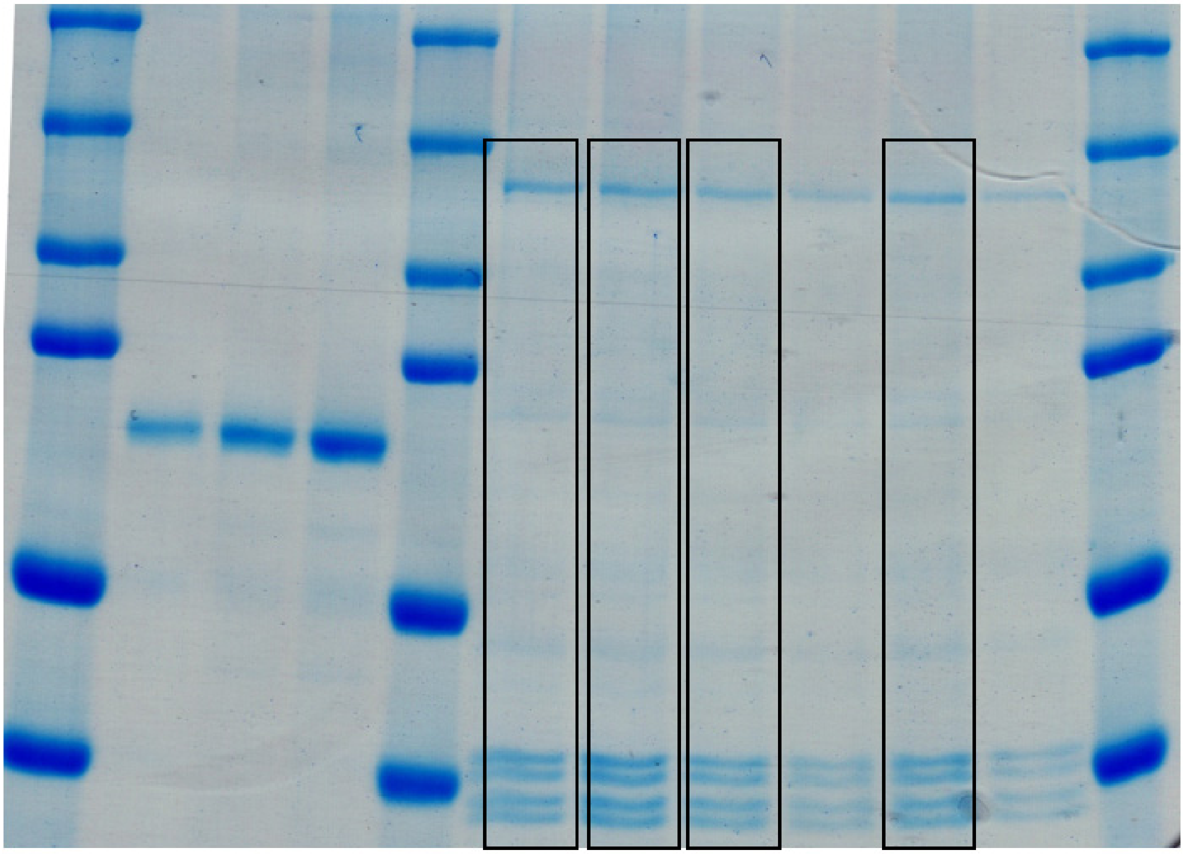

**Figure 3C**

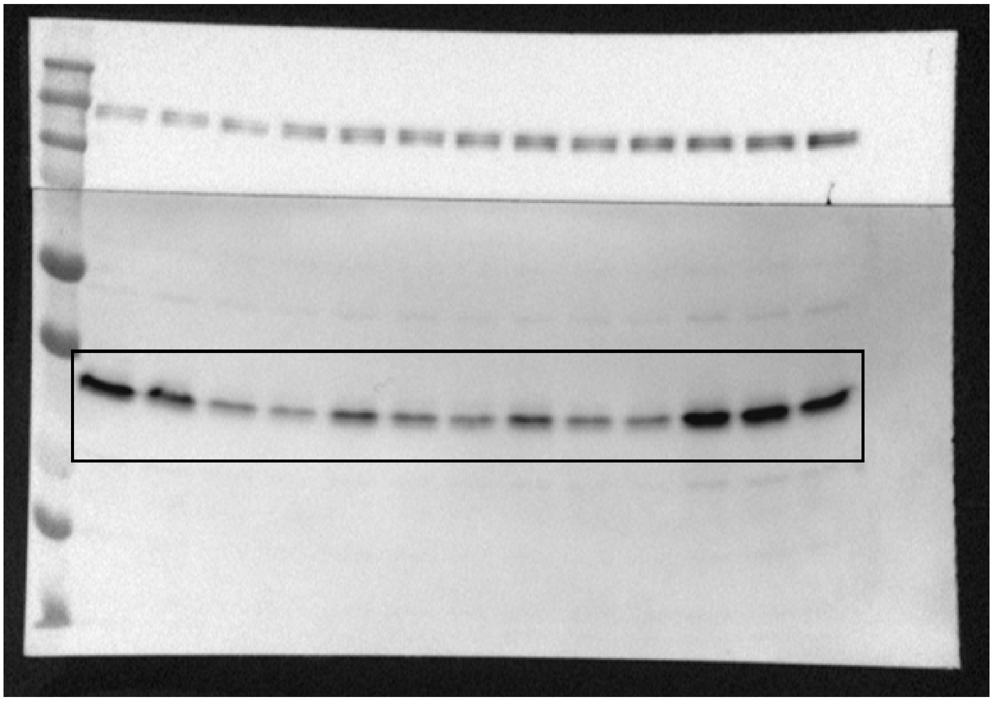

PCNA

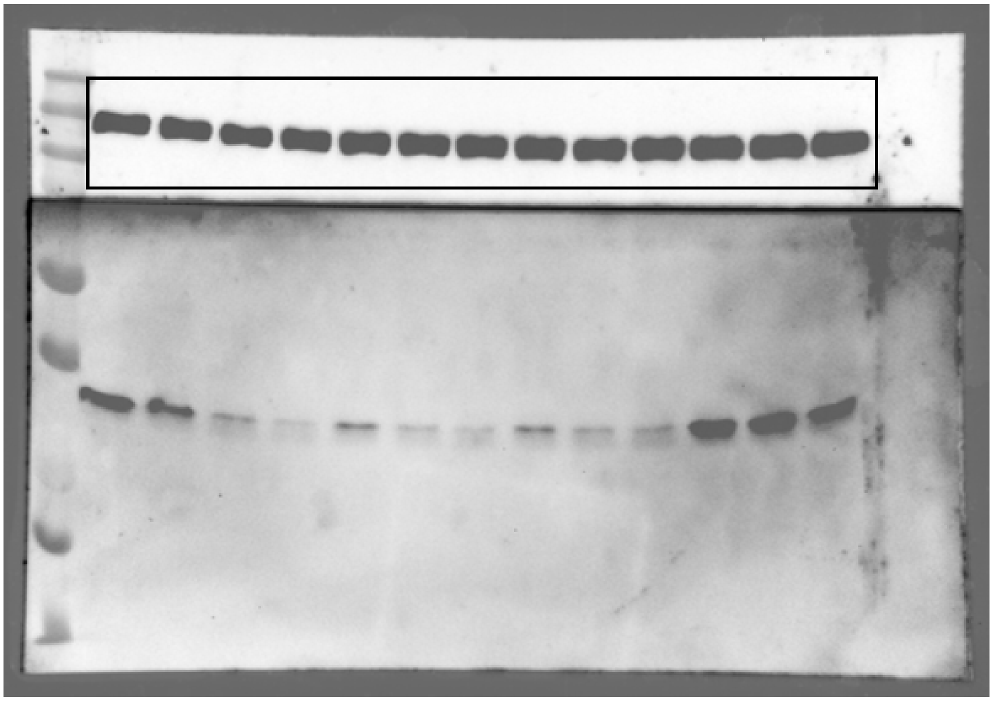

TALE (FLAG)

**Figure 3C_repeat**

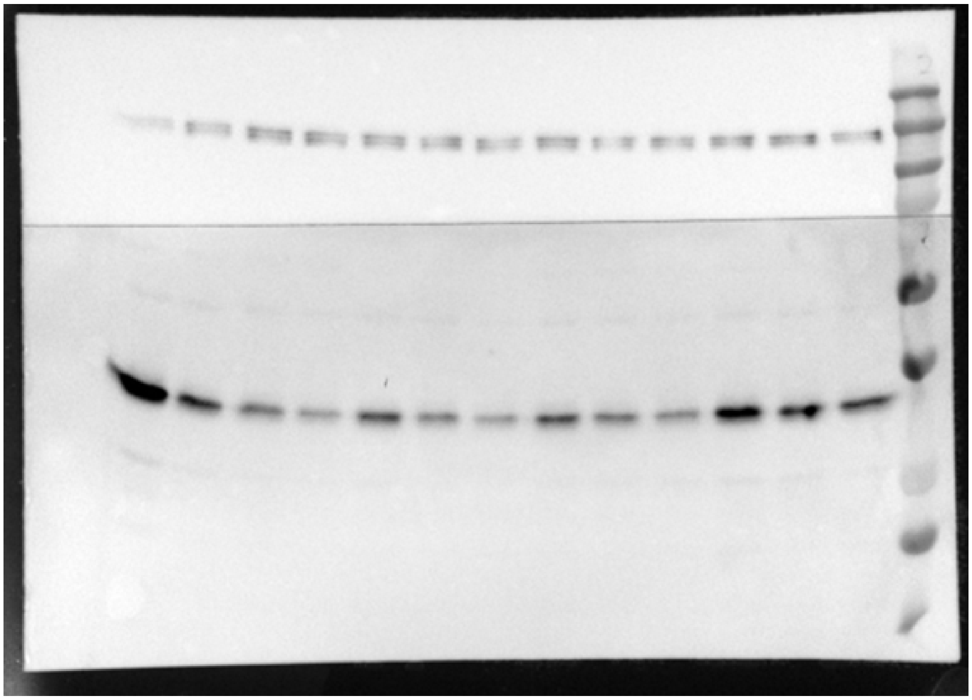

PCNA

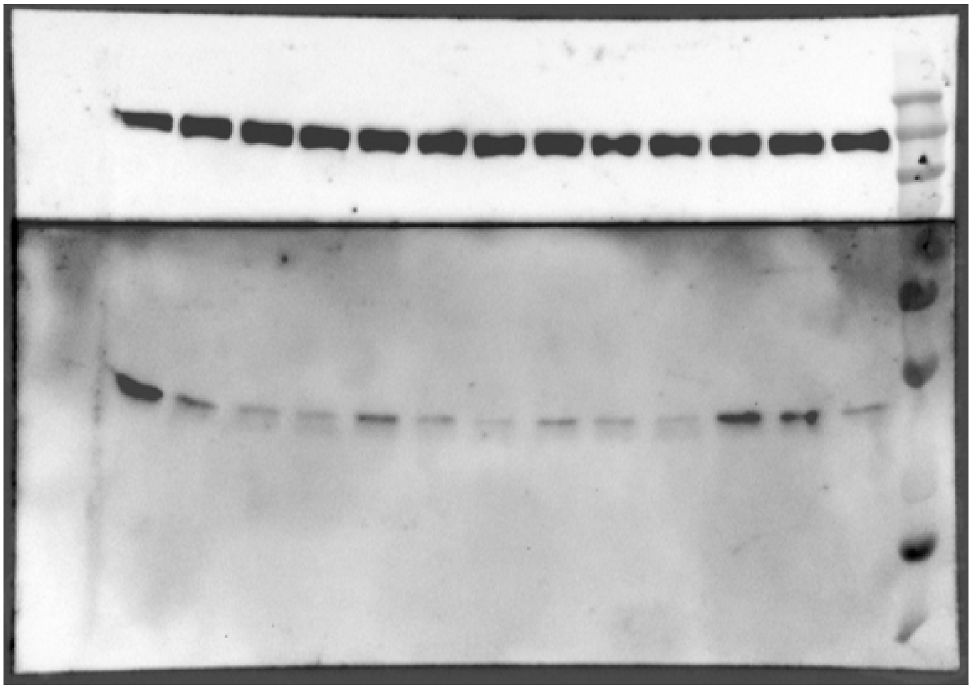

TALE (FLAG)

**Figure 4A**

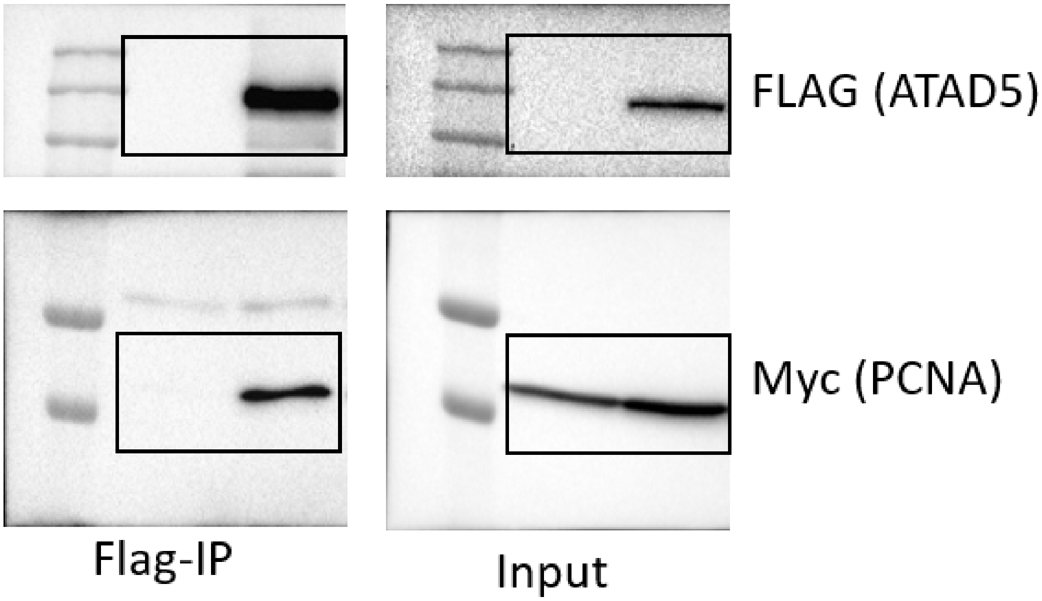

**Figure 4B**

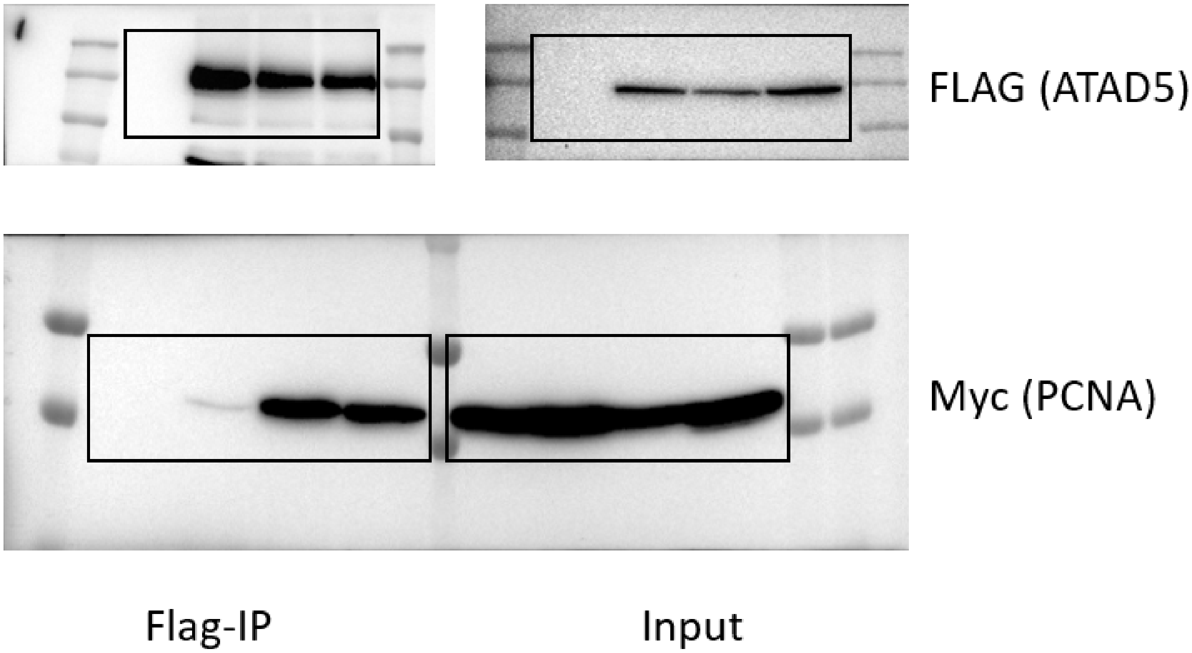

**Figure 4C**

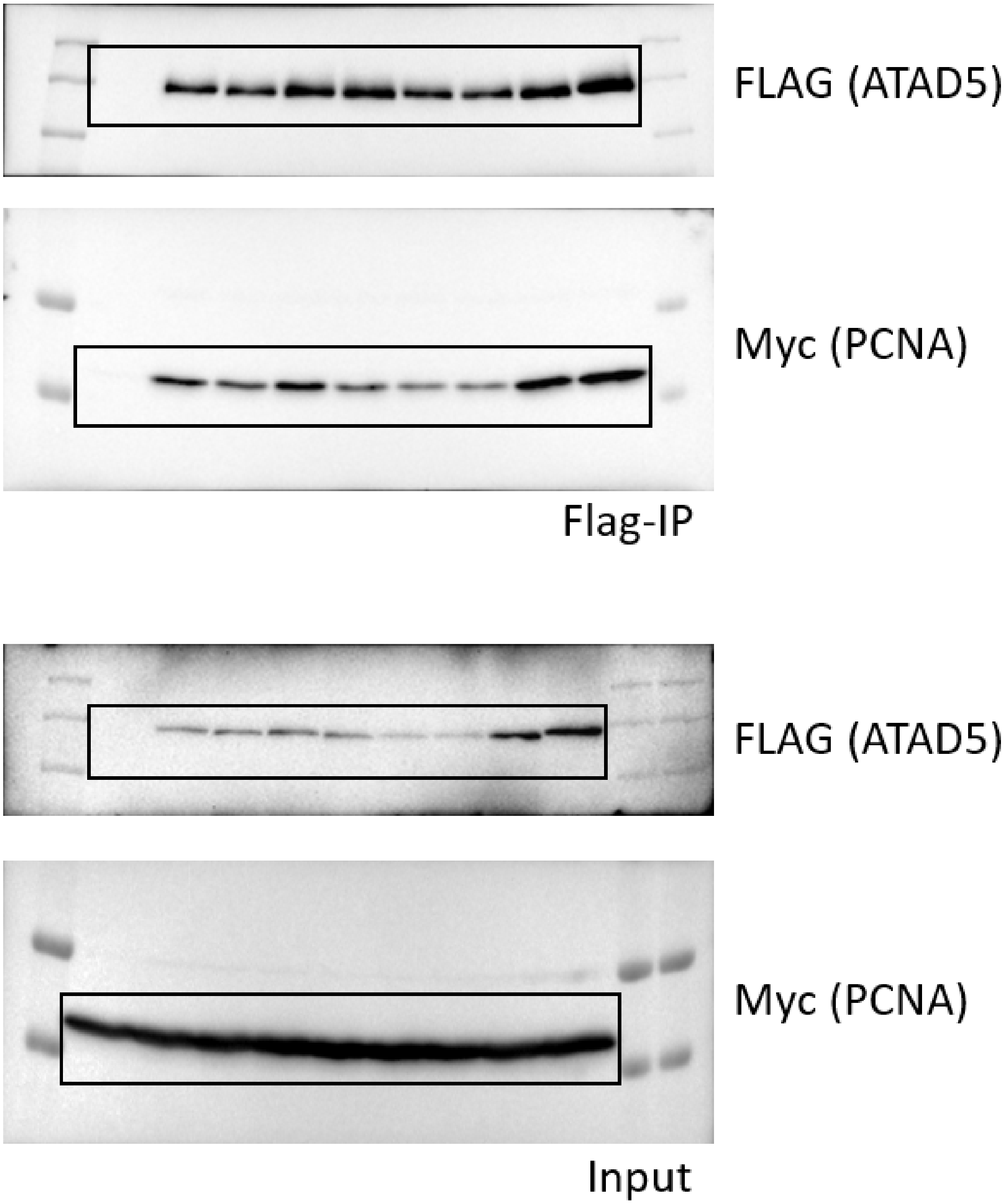

**Figure 4C_repeat**

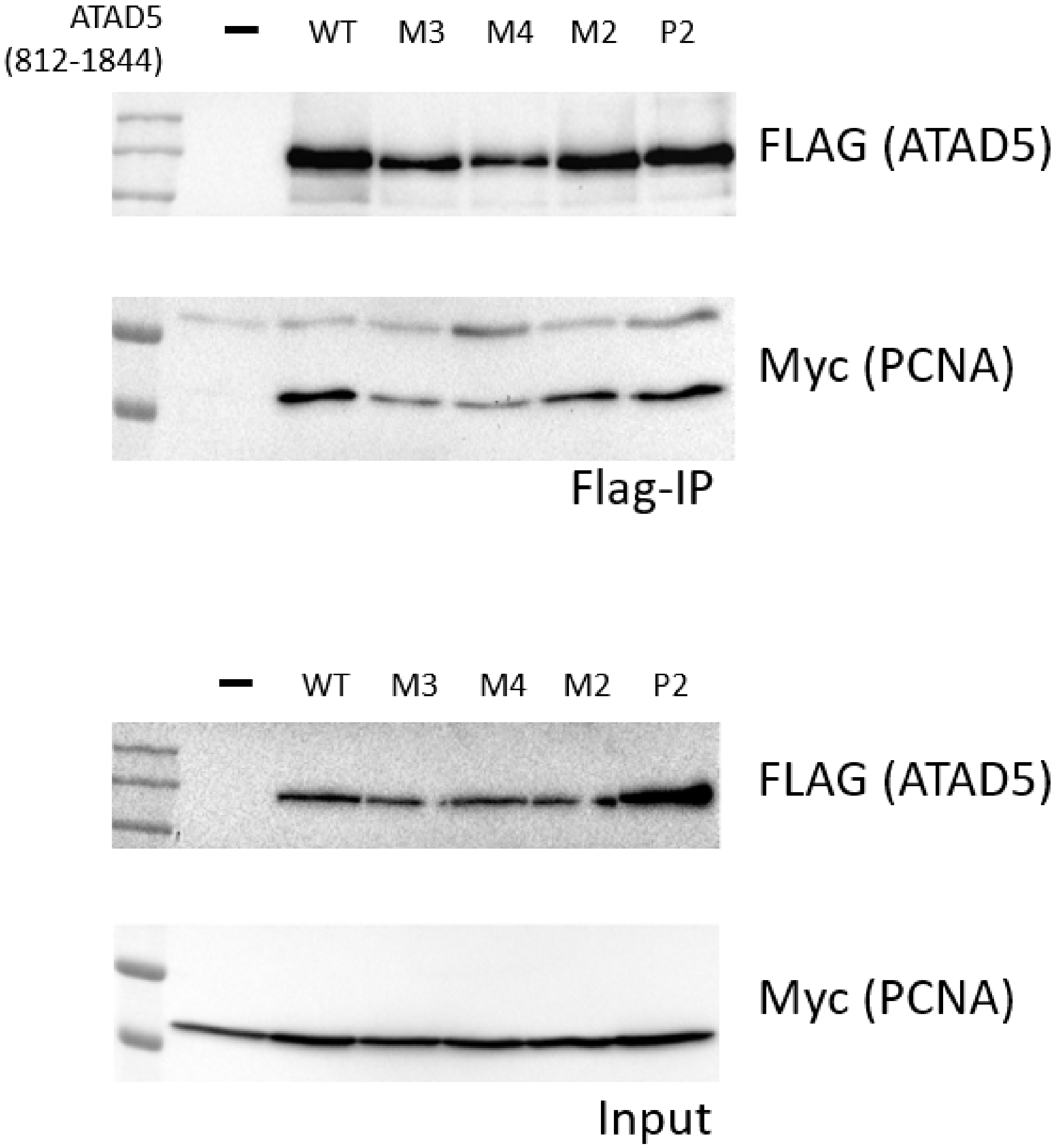

**Figure 4D**

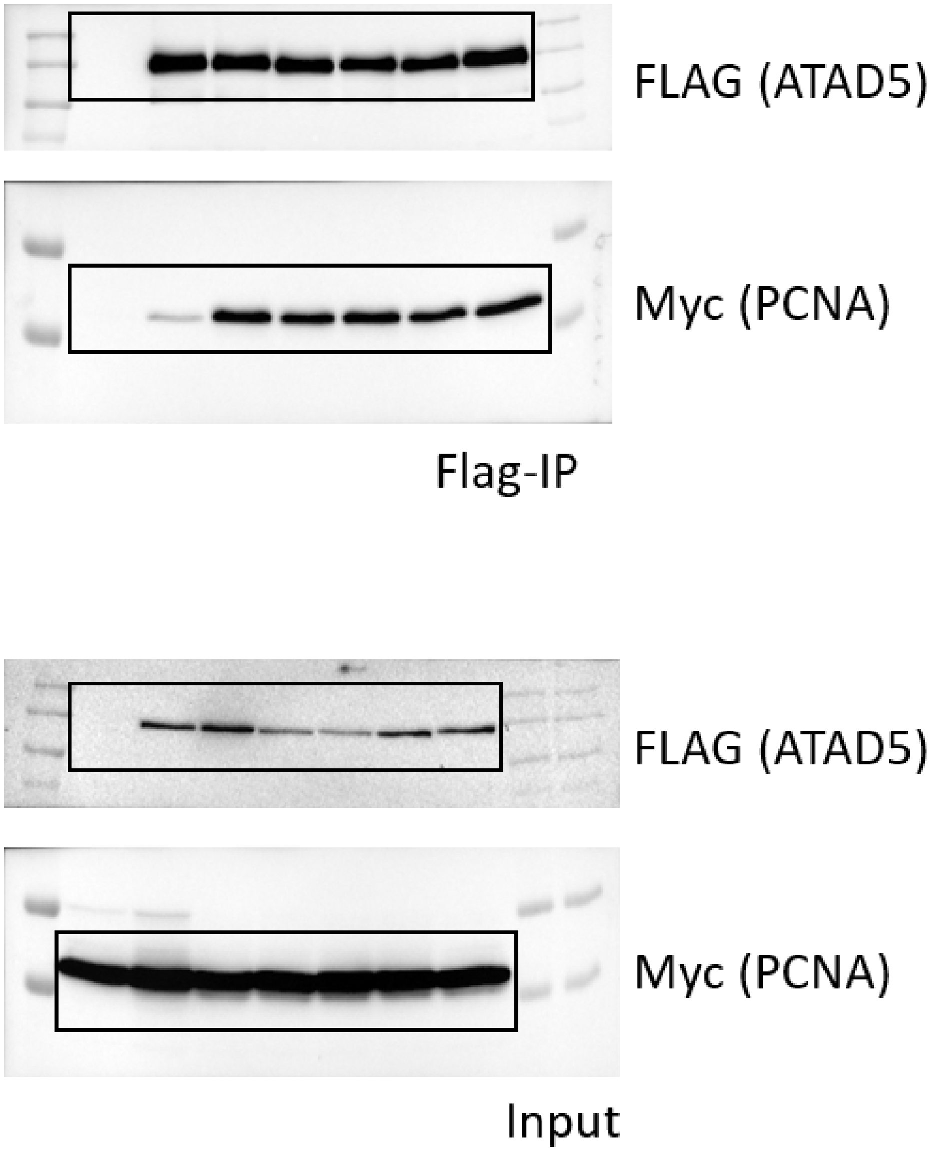

**Figure 4D_repeat**

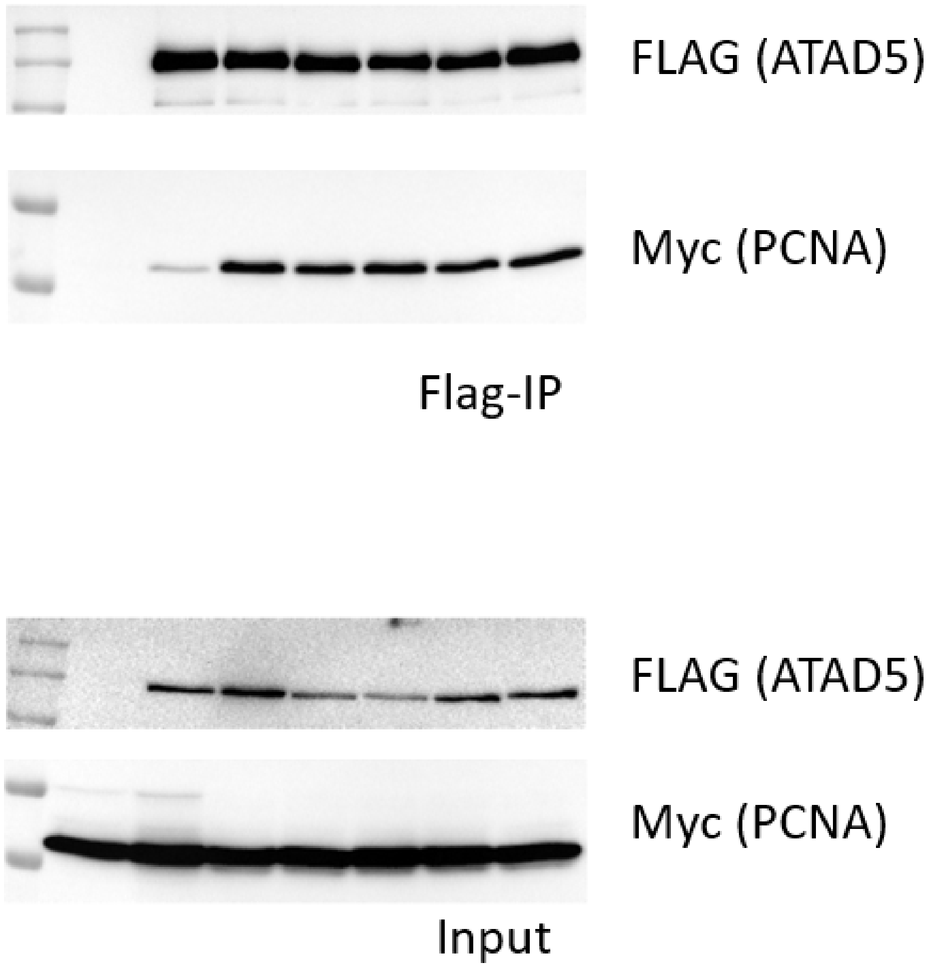

**Figure 5A**

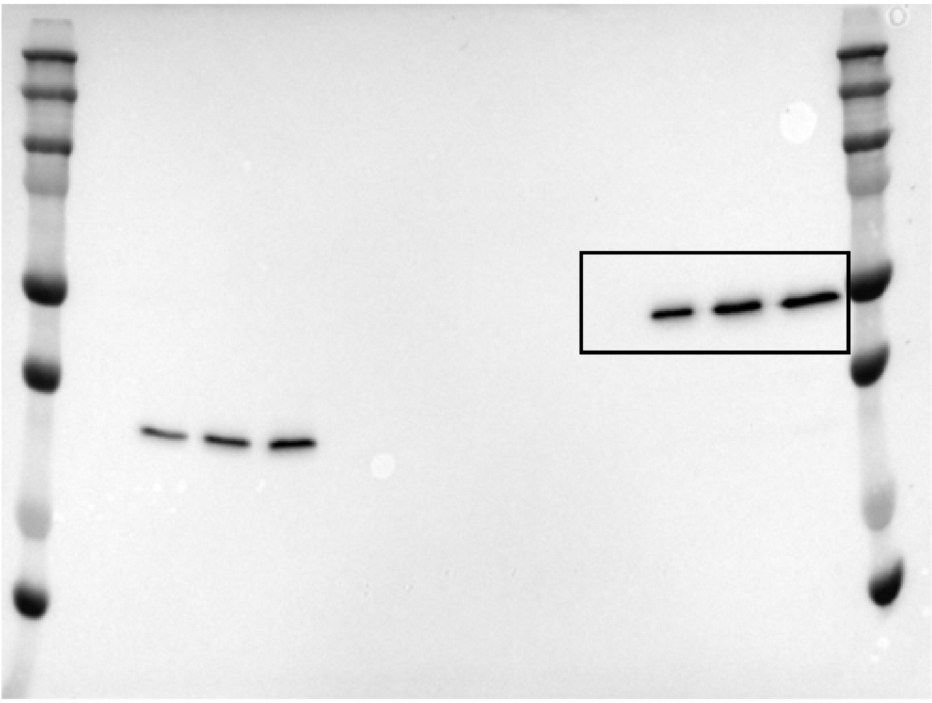

**Figure 5B**

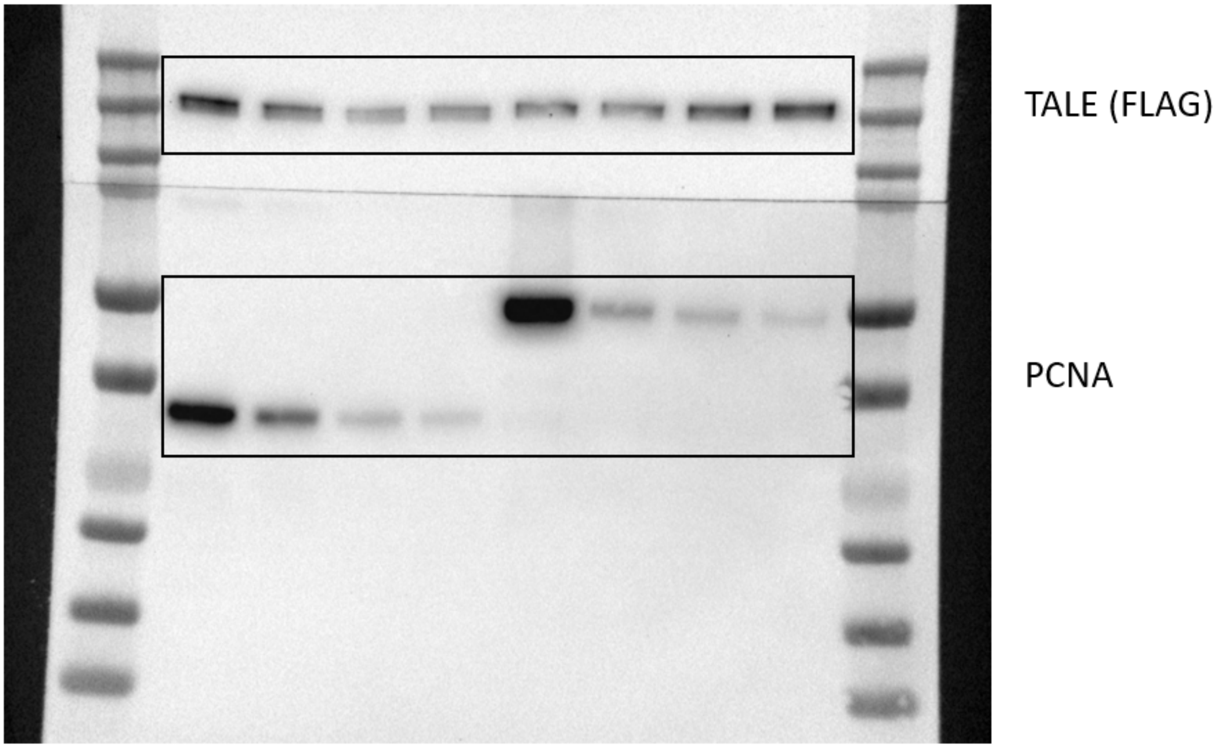

**Figure 5B_repeat**

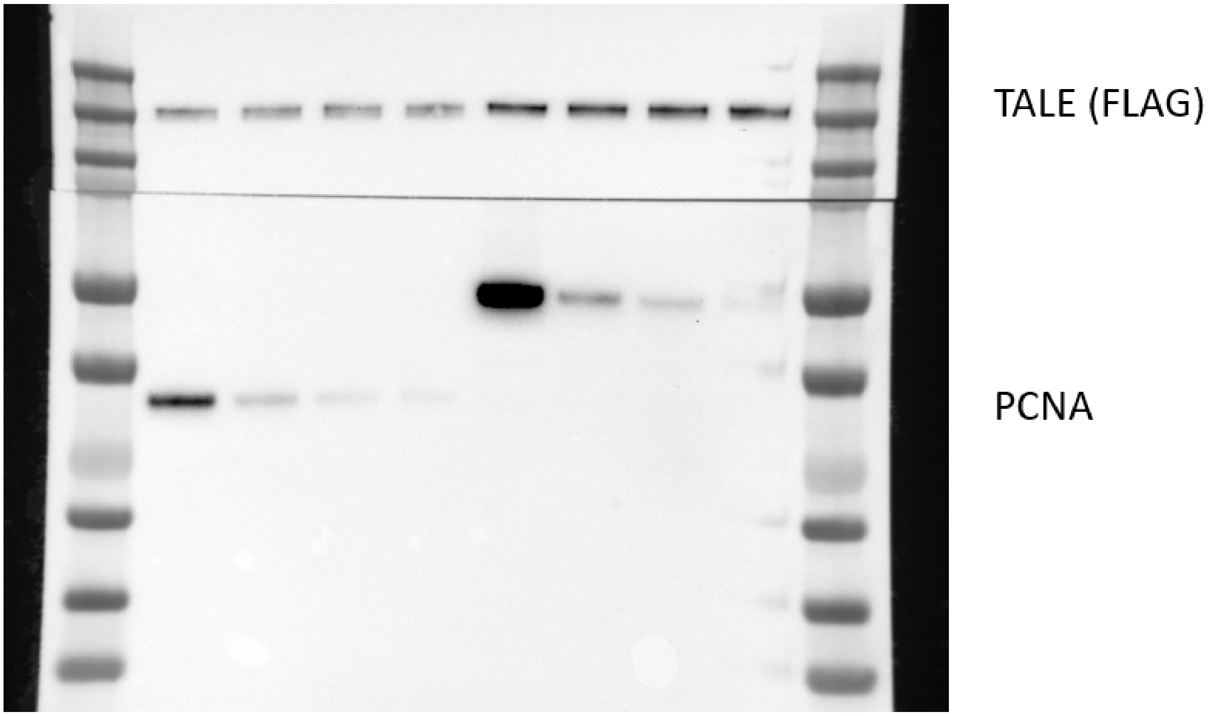

**Figure 5C**

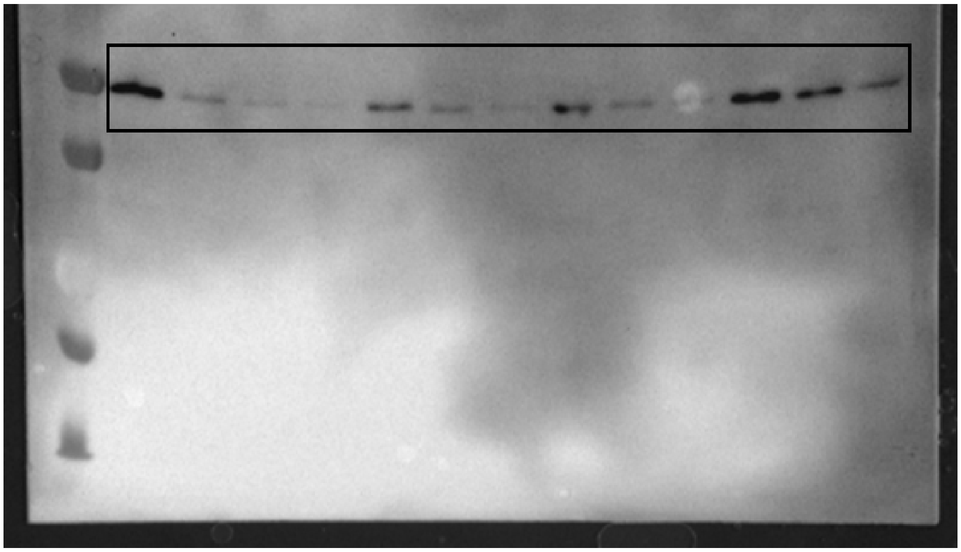

PCNA

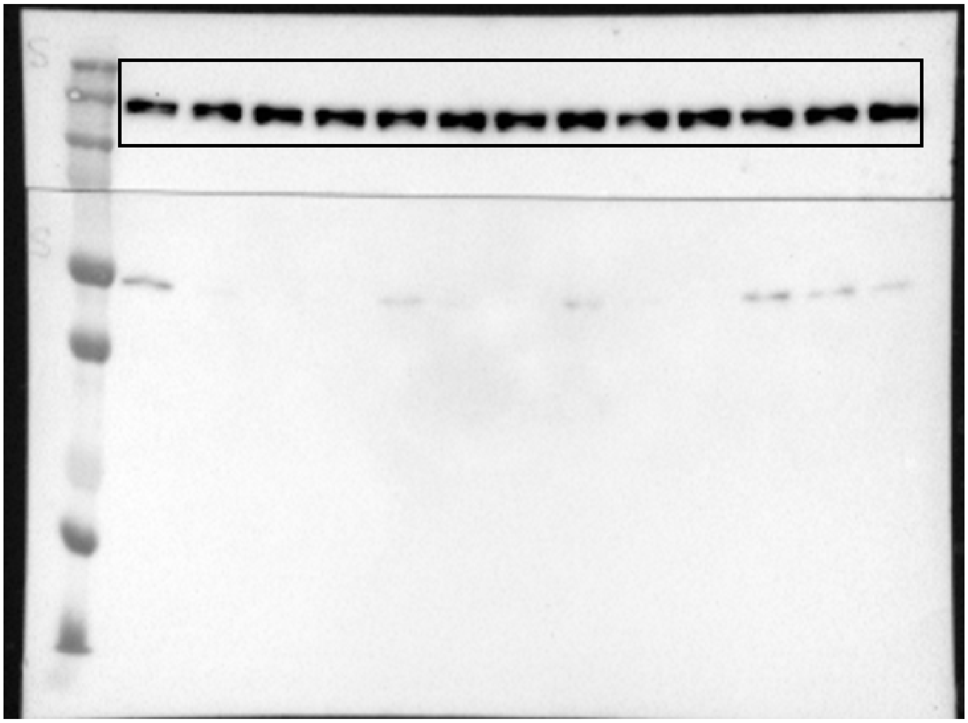

TALE (FLAG)

**Figure 5C_repeat**

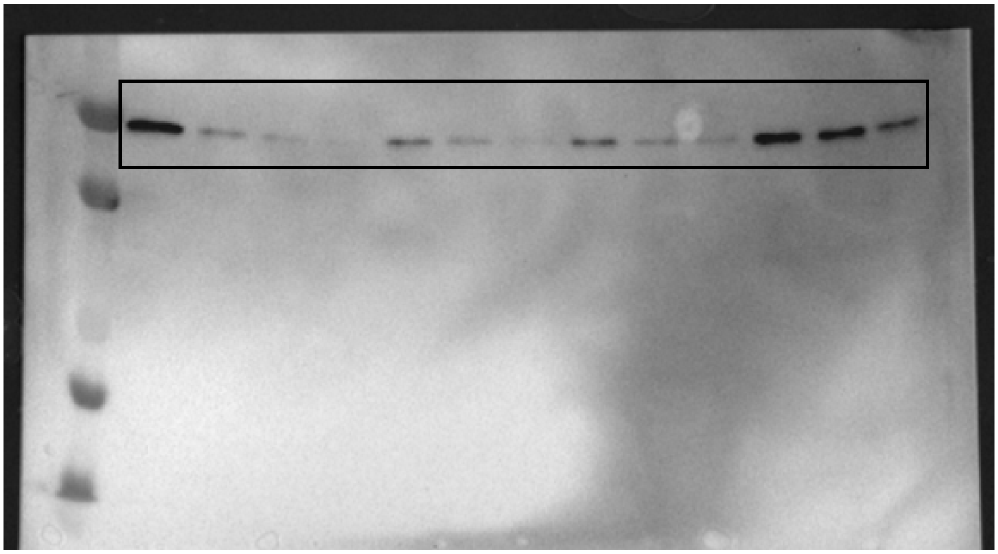

PCNA

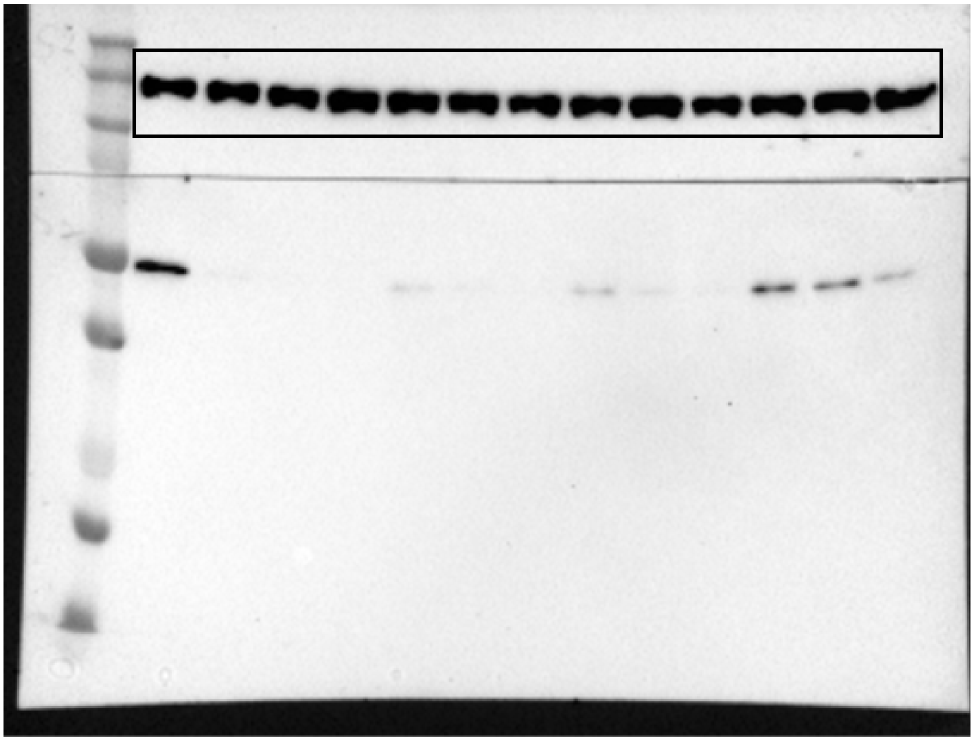

TALE (FLAG)

**Figure 6B**

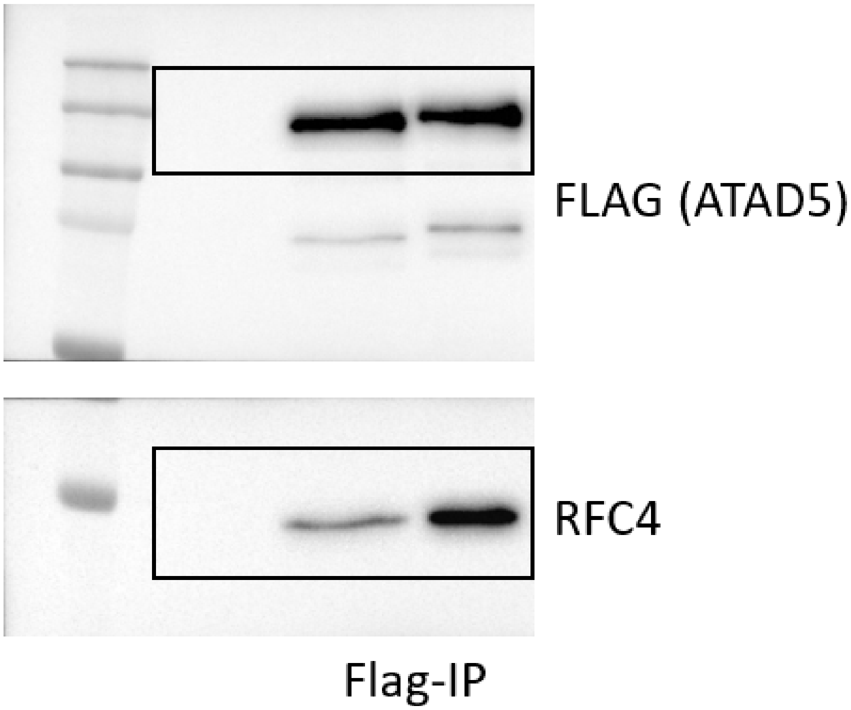

**Figure 6C**

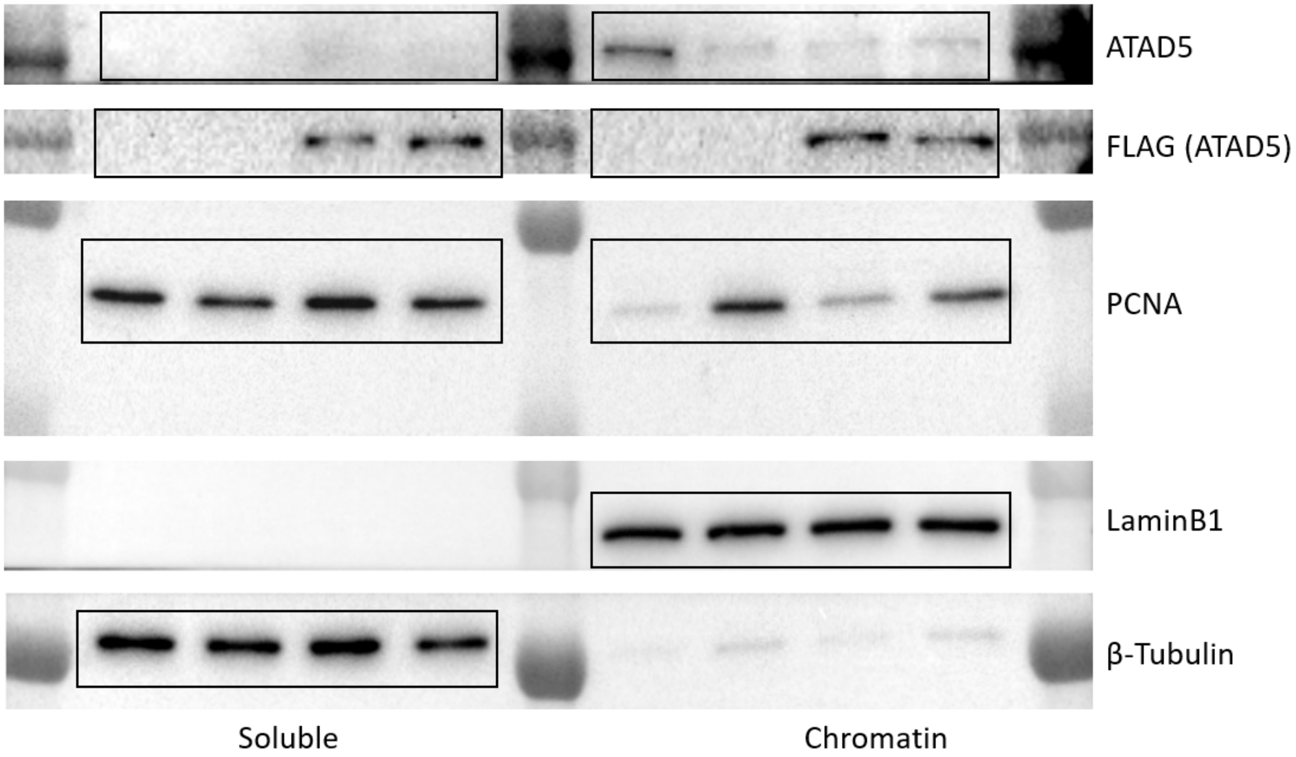

**Figure 6C_repeat**

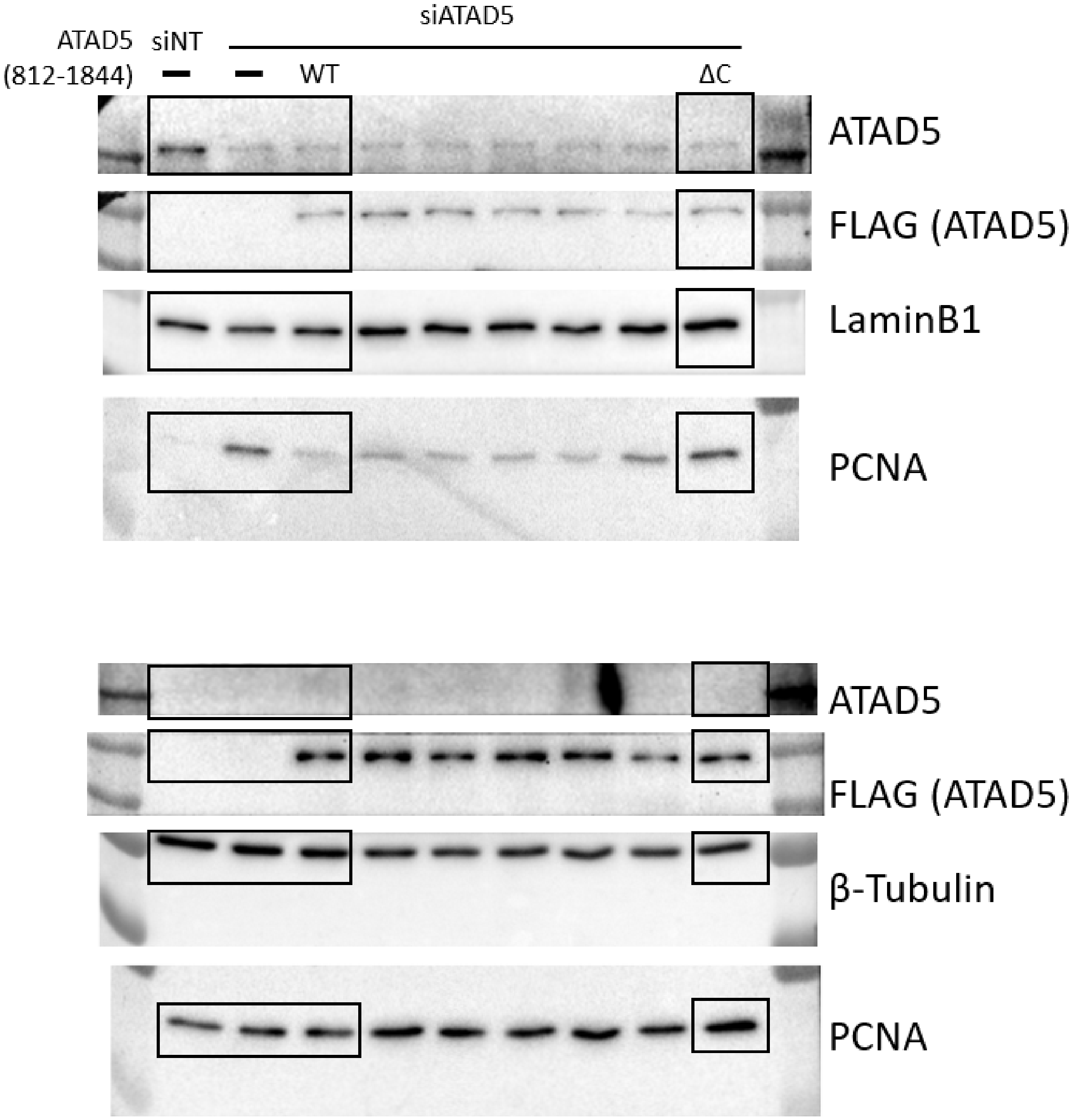

**Figure S1A**

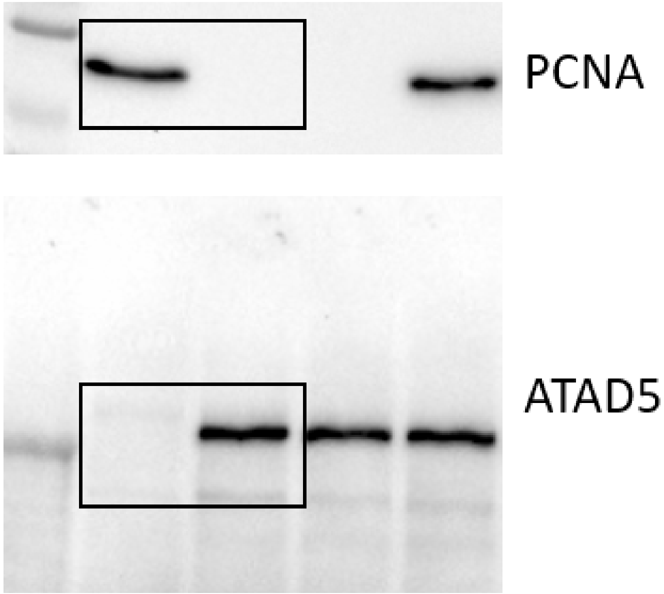

**Figure S2B**

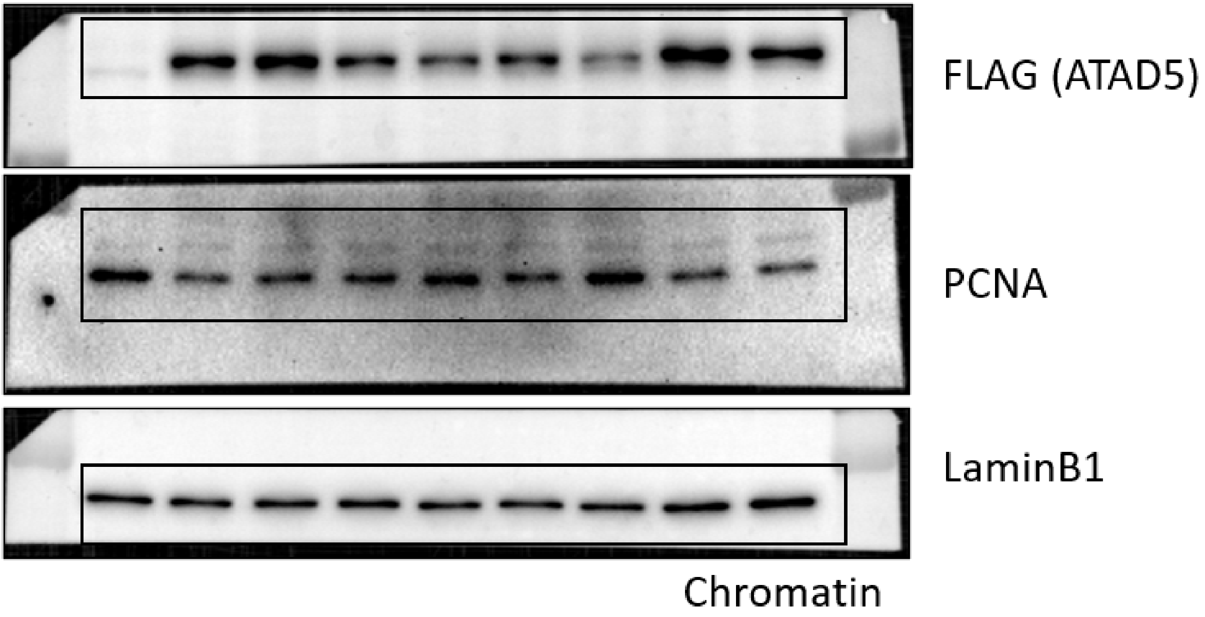
FLAG (ATAD5)
PCNA
LaminB1
Chromatin

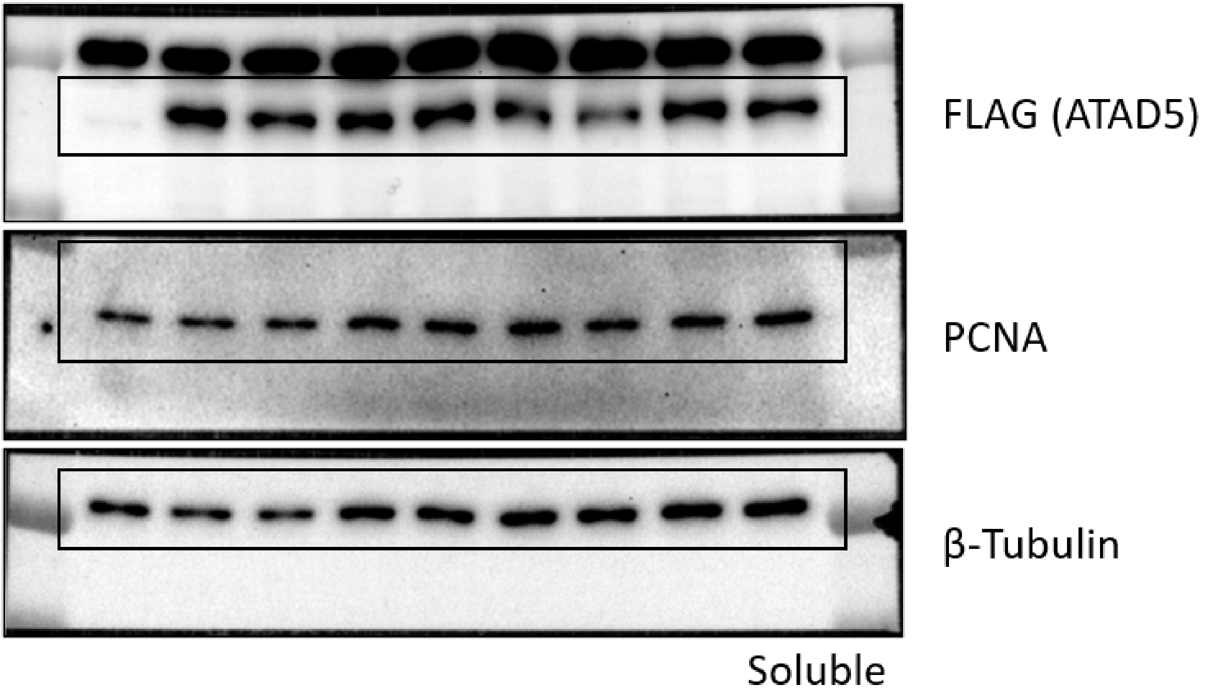
FLAG (ATAD5)
PCNA
β-Tubulin
Soluble

**Figure S2B_repeat**

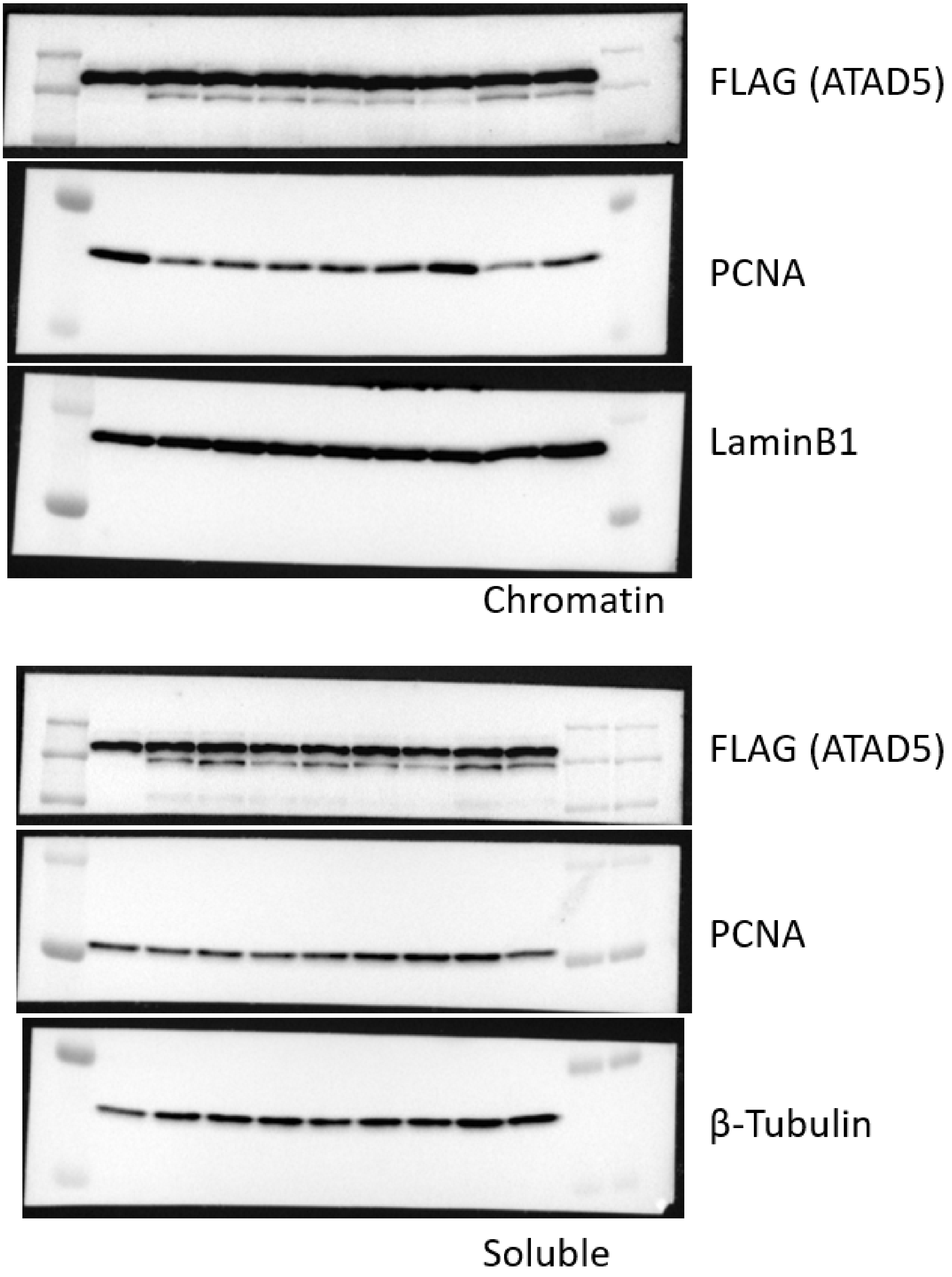


**Figure S3A**

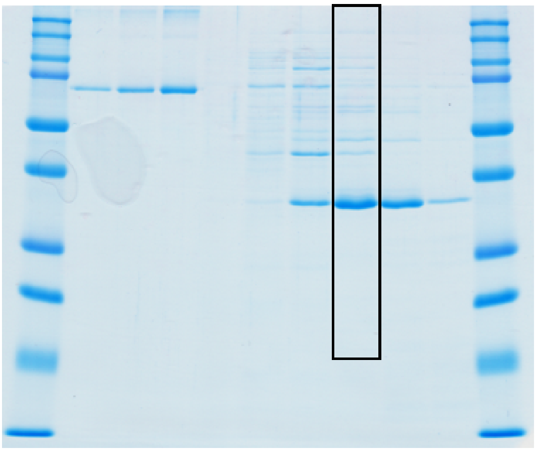

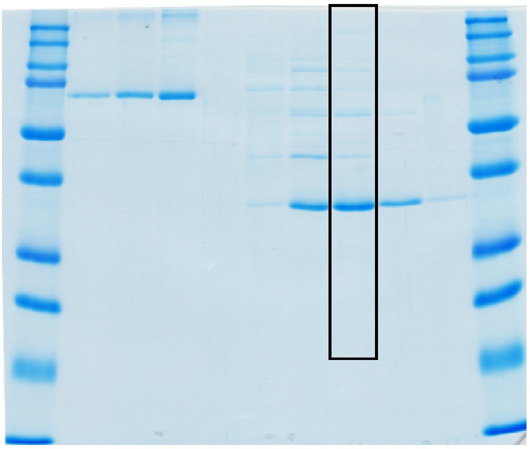

**Figure S3B**

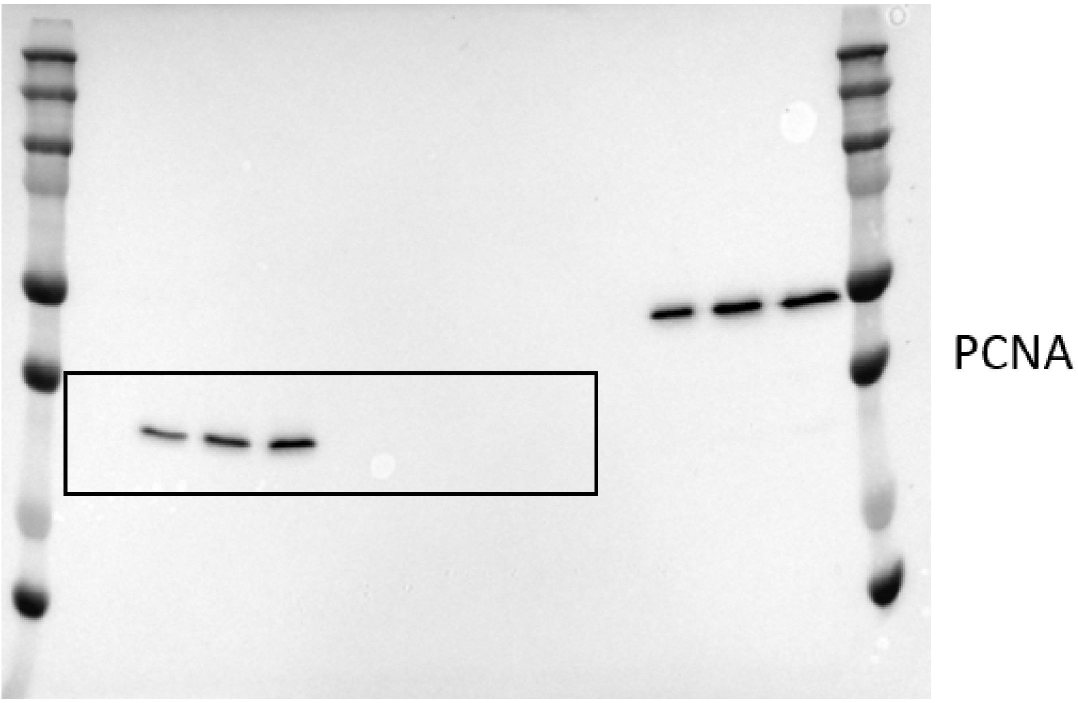

**Figure S3C**

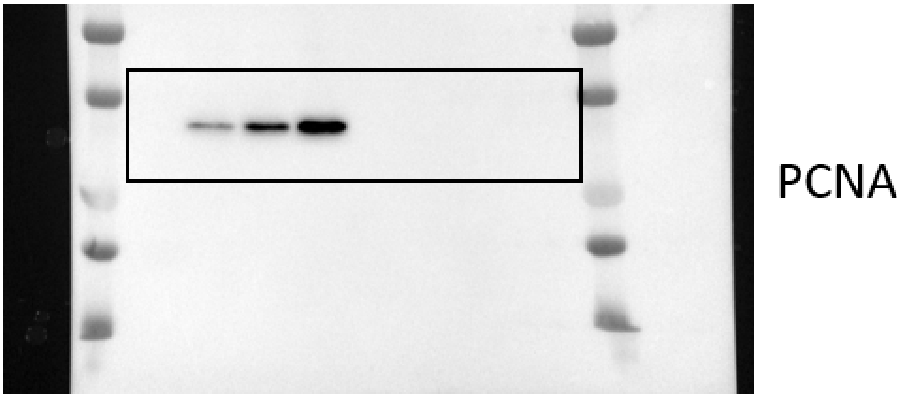

**Figure S3D**

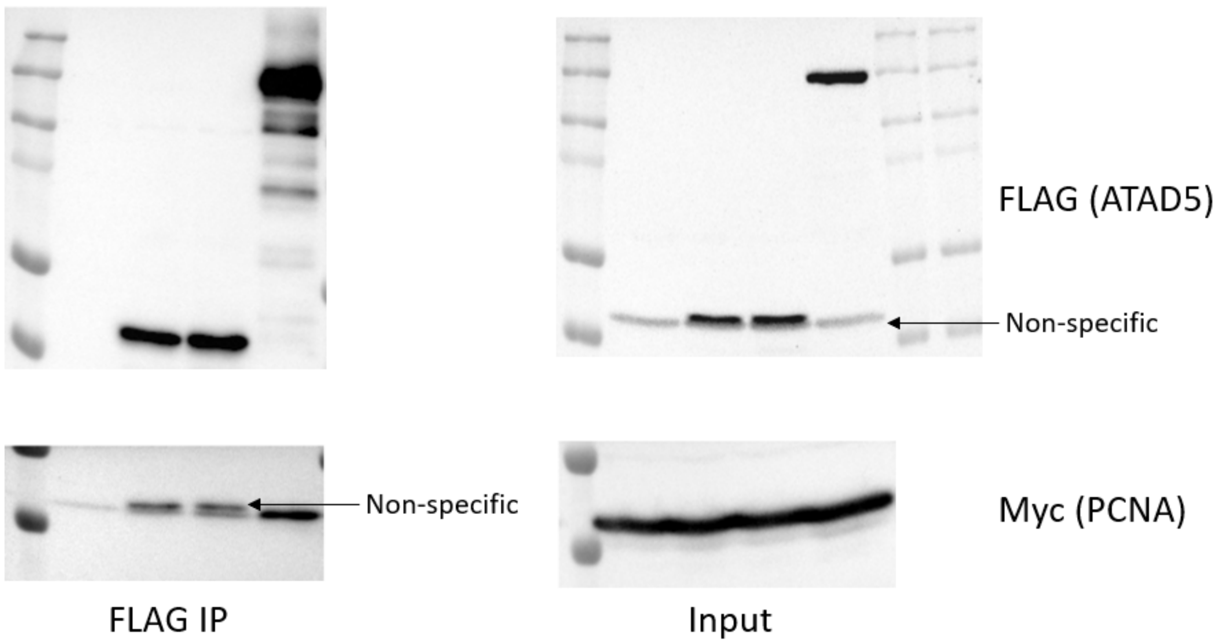

**Figure S3D_repeat**

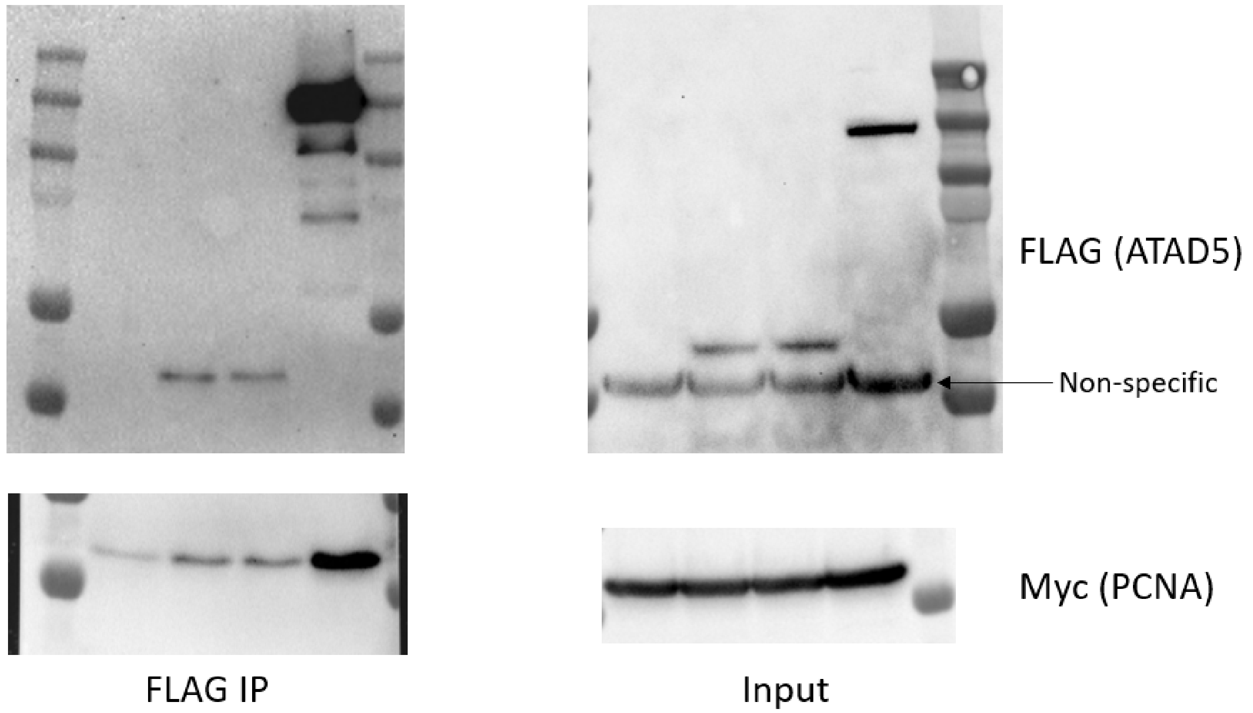

**Figure S3E**

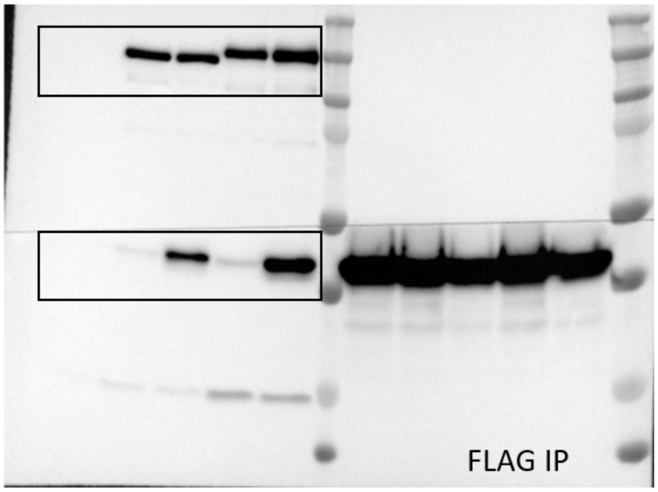

FLAG (ATAD5)

Myc (PCNA)

FLAG IP

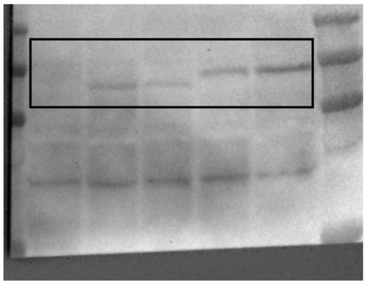

FLAG (ATAD5)

Input

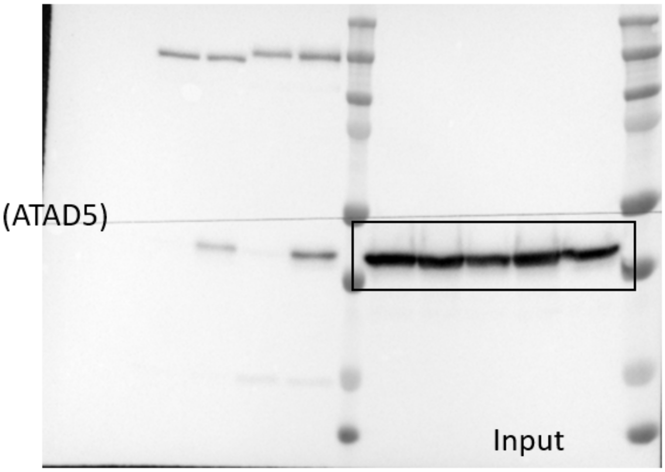

Myc (PCNA)

Input

**Figure S3E_repeat**

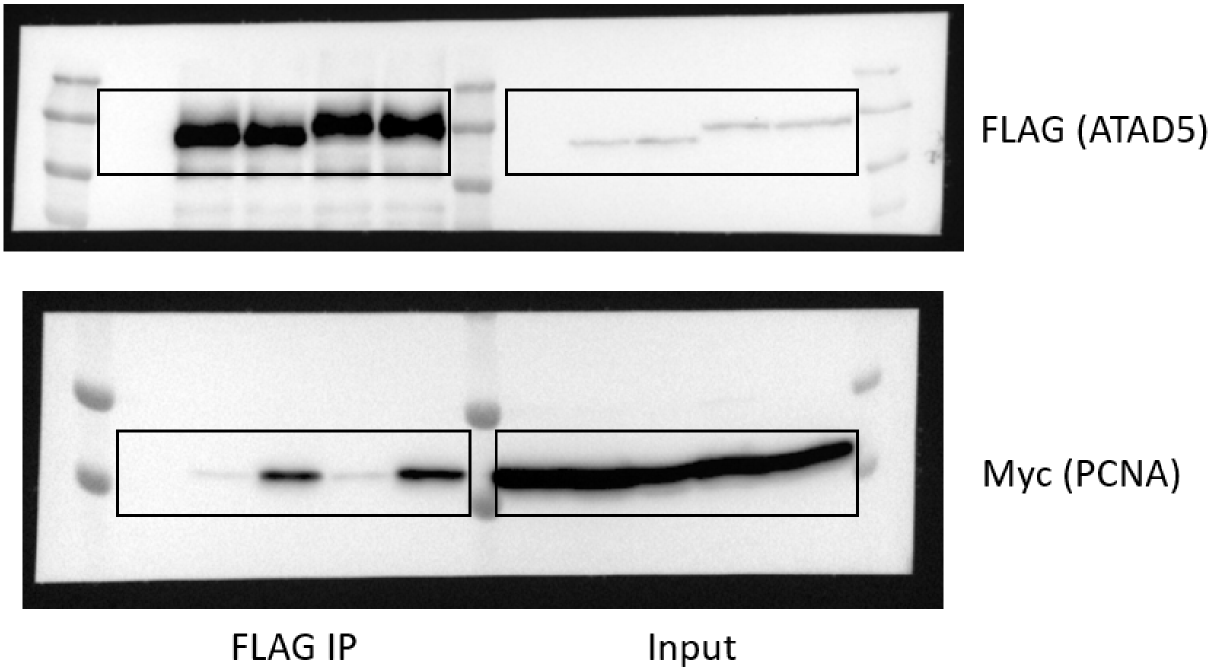

**Figure S4A**

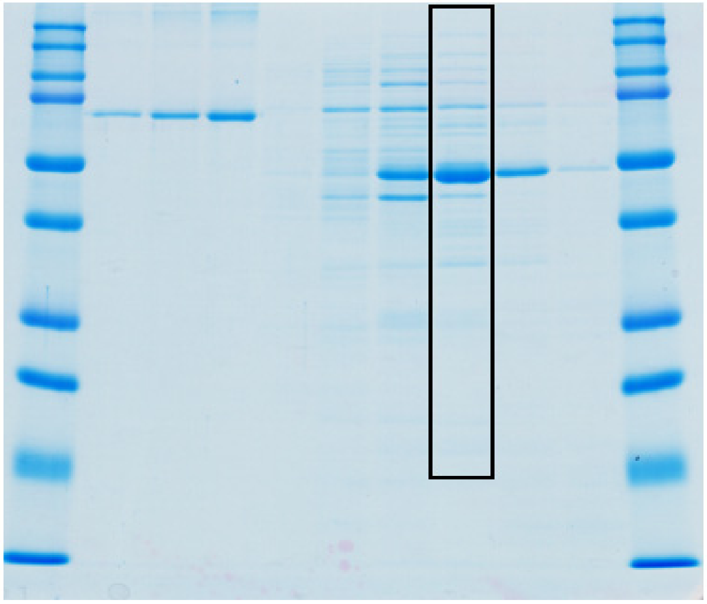

**Figure S4B**

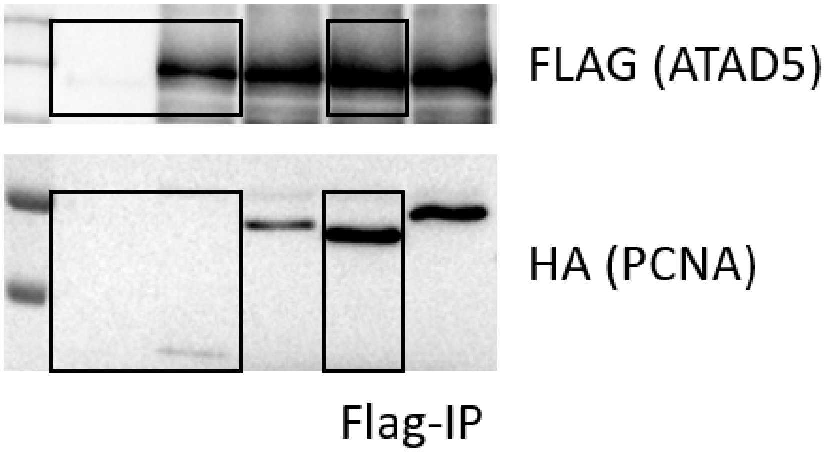


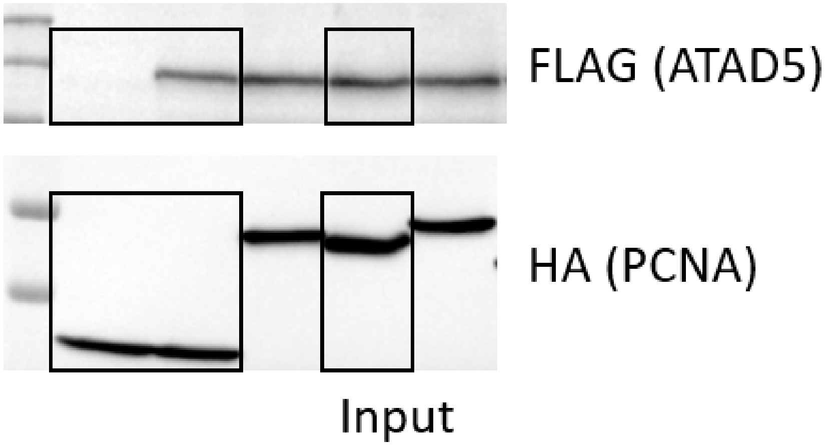

**Figure S4C**

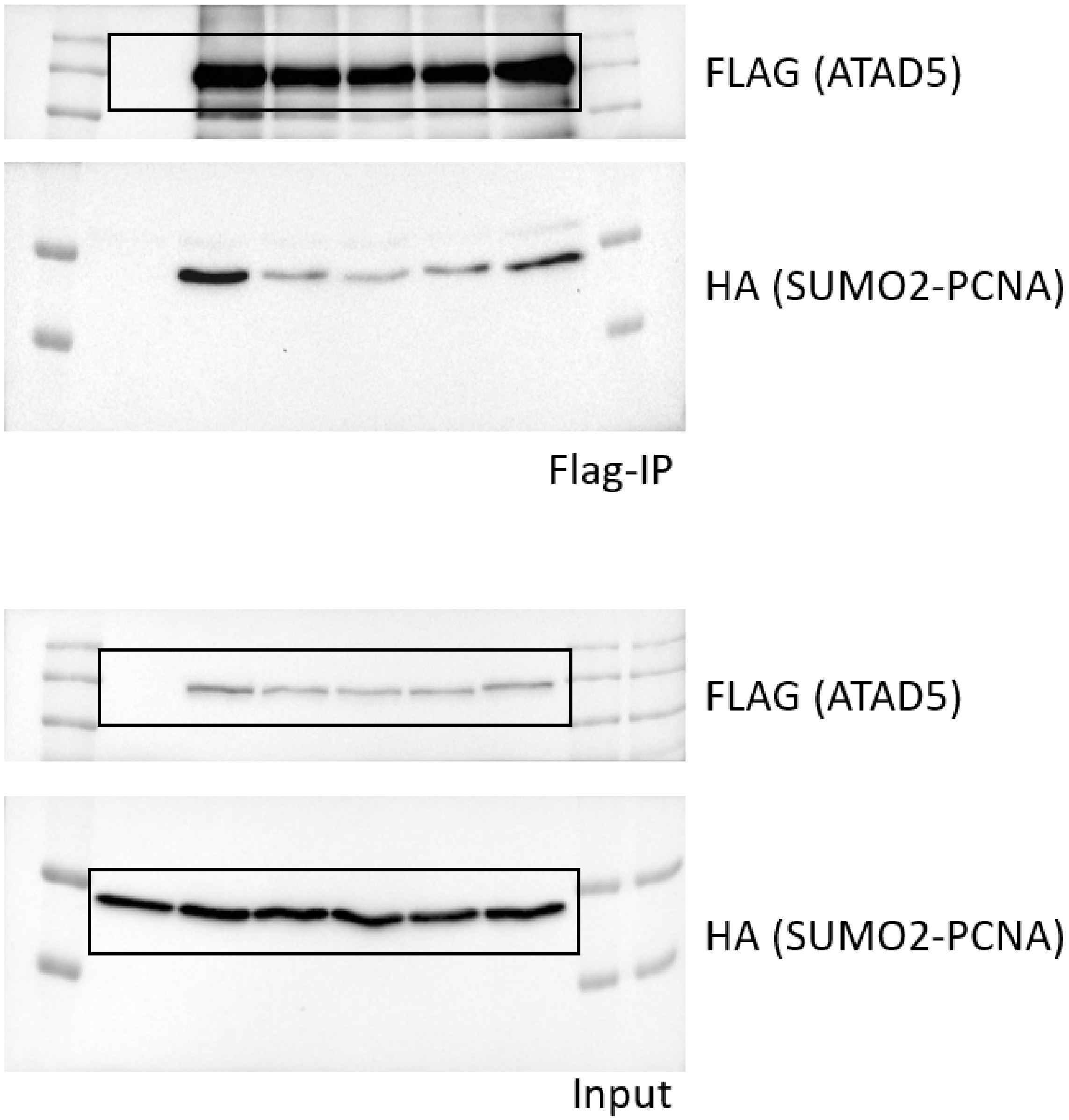
FLAG (ATAD5)
HA (SUMO2-PCNA)
Flag-IP
FLAG (ATAD5)
HA (SUMO2-PCNA)
Input

**Figure S4C_repeat**

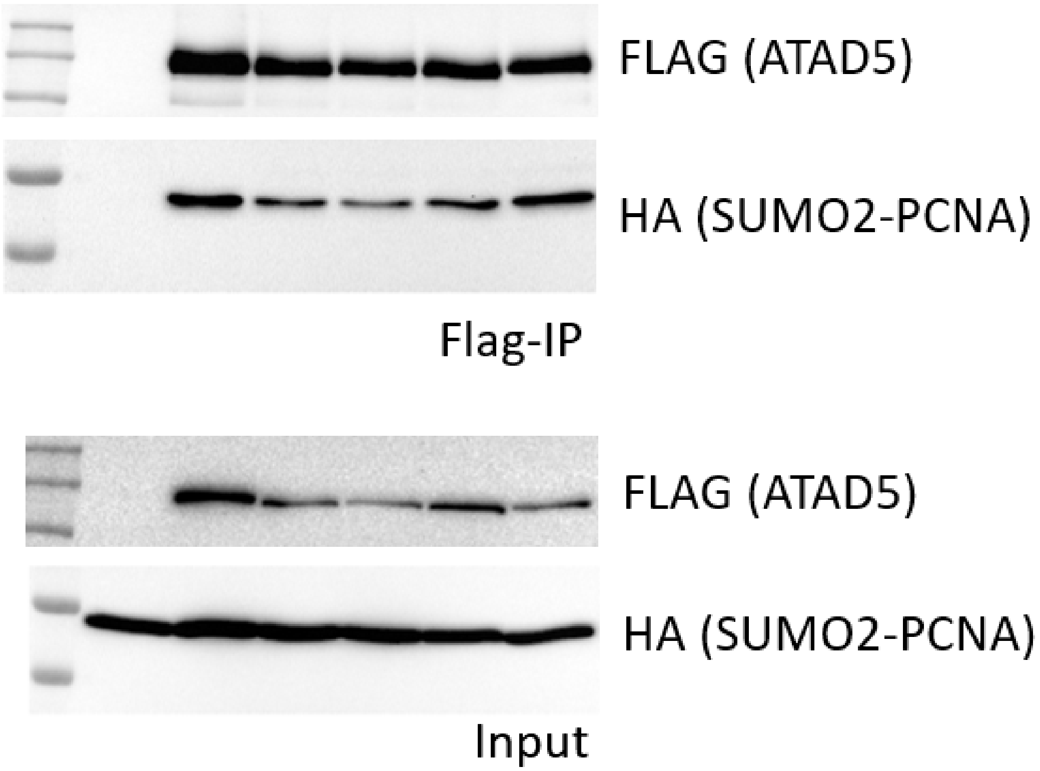

Supplement: Supplementary file 1 [file cells-11-01832-s001.zip › cells-1706334-supplementary.pdf]
